# A multivariant recall‐by‐genotype study of the metabolomic signature of BMI

**DOI:** 10.1002/oby.23441

**Published:** 2022-05-22

**Authors:** Si Fang, Kaitlin H. Wade, David A. Hughes, Sophie Fitzgibbon, Vikki Yip, Nicholas J. Timpson, Laura J. Corbin

**Affiliations:** ^1^ MRC Integrative Epidemiology Unit at the University of Bristol Bristol UK; ^2^ Population Health Science Bristol Medical School University of Bristol Bristol UK; ^3^ Bristol Bioresource Laboratories Population Health Science Bristol Medical School University of Bristol Bristol UK

## Abstract

**Objective:**

This study estimated the effect of BMI on circulating metabolites in young adults using a recall‐by‐genotype study design.

**Methods:**

A recall‐by‐genotype study was implemented in the Avon Longitudinal Study of Parents and Children. Samples from 756 participants were selected for untargeted metabolomics analysis based on low versus high genetic liability for higher BMI defined by a genetic risk score (GRS). Regression analyses were performed to investigate associations between BMI GRS group and relative abundance of 973 metabolites.

**Results:**

After correction for multiple testing, 29 metabolites were associated with BMI GRS group. Bilirubin was among the most strongly associated metabolites, with reduced levels measured in individuals in the high‐BMI GRS group (*β* = −0.32, 95% CI: −0.46 to −0.18, Benjamini‐Hochberg adjusted *p* = 0.005). This study observed associations between BMI GRS group and the levels of several potentially diet‐related metabolites, including hippurate, which had lower mean abundance in individuals in the high‐BMI GRS group (*β* = −0.29, 95% CI: −0.44 to −0.15, Benjamini‐Hochberg adjusted *p* = 0.008).

**Conclusions:**

Together with existing literature, these results suggest that a genetic predisposition to higher BMI captures differences in metabolism leading to adiposity gain. In the absence of prospective data, separating these effects from the downstream consequences of weight gain is challenging.

**Study Importance**

**What is already known?**

► Metabolomics, defined as the measurement and study of circulating small molecules that are the substrates and products of cellular metabolism, is increasingly used by epidemiologists to provide a functional readout of bulk cellular activity and a proxy for individual current health. This approach also provides insight into biological pathways linking exposure and disease.
► In observational studies, elevated BMI has been associated with a wide range of circulating metabolites. Researchers are now looking to genetic epidemiological methods, such as Mendelian randomization, to offer insight into potential causal relationships.

**What does this study add?**

► We identified 29 metabolites for which relative abundance varies with a genetic predisposition to higher BMI.
► Bilirubin, a key component of the heme catabolic pathway and a potent antioxidant, showed the strongest association.

**How might these results change the direction of research or the focus of clinical practice?**

► Results of both Mendelian randomization and recall‐by‐genotype studies need to be combined with alternative study designs to distinguish biomarkers that are intermediates on the pathway to BMI from those reflective of metabolic changes that result from increased BMI.
► Separating causal from noncausal biomarkers of adiposity is important because only the former are relevant to treatment and prevention, whereas both could be informative with respect to prediction and the downstream consequences of high BMI.

## INTRODUCTION

Despite the extensive focus in the literature, the full downstream impact of high BMI and the potential causal mechanisms by which BMI impacts a large number of noncommunicable diseases remain unclear (1). Through a combination of large‐scale observational studies and intervention designs, BMI has been established as a major risk factor for many common complex diseases, including type 2 diabetes mellitus, hypertension, myocardial infarction, stroke, and cancer (2, 3, 4). Although the precision of effect estimates describing the association between BMI and disease (and our confidence in them) has increased with greater sample sizes and independent replication, observational studies are limited by confounding, bias, and reverse causation. Meanwhile, intervention studies (e.g., weight‐change protocols) designed to circumvent these conventional limitations have their own challenges, notably a limited ability to alter BMI to the extent required to quantify an effect and the necessarily short‐term and small‐scale nature of such interventions.

In response to these challenges, and following developments in understanding genetic contributions to BMI, methods from applied genetic epidemiology are now being used to dissect the relationship between BMI and health. One approach in which genetic variants act as an approximation to instrumental variables to evaluate the causal effect of an exposure (adiposity) on an outcome (disease) is Mendelian randomization (MR). In MR, genetic variation fulfills the role of an instrumental variable (5), in which the presence of variance in BMI explained by genotype is (in principle) orthogonal to confounding factors and genotype is assumed to exert an effect on health outcome only through BMI. Despite validating the likely causal nature of the relationship observed between BMI and disease, these studies do little to explain *how* risk is delivered.

Metabolomics uses various techniques to measure low‐molecular‐weight metabolites across body fluids and tissues and can be used to provide a functional readout of an individual’s health. Its use in epidemiological studies is increasing and it has the potential to help elucidate the mechanisms linking obesity and associated comorbidities, as well as to identify biomarkers to facilitate intervention and treatment. To date, studies have shown BMI‐associated changes across a range of metabolite classes, including sex steroids, branched‐chain and aromatic amino acids, acylcarnitines, and lipids (6). But with much of the existing literature on the metabolomic impact of adiposity being based on observational analyses, gaps remain in our understanding of the biology underpinning the development and direct pathophysiological consequences of obesity.

We aimed to integrate the use of genetic predictors for BMI with in‐depth intermediate phenotyping to explore the relationship between BMI and metabolic health. Recall by genotype (RbG) is a study design in which participants (or samples) are selected from a preexisting cohort based on genetic variation either at single variants or in the form of a genetic risk score (GRS) (7). In this way, RbG exploits the concept of MR (i.e., the random assortment of genetic variants in offspring), enables greater power for a given number of samples analyzed as compared with random selection, facilitates deep phenotyping, and is less prone to confounding and reverse causality (7). The aim of this RbG study was to examine the effect of BMI (the exposure) on circulating metabolites (the outcome) using a GRS describing a high versus low predisposition to higher BMI.

## METHODS

**FIGURE 1:**
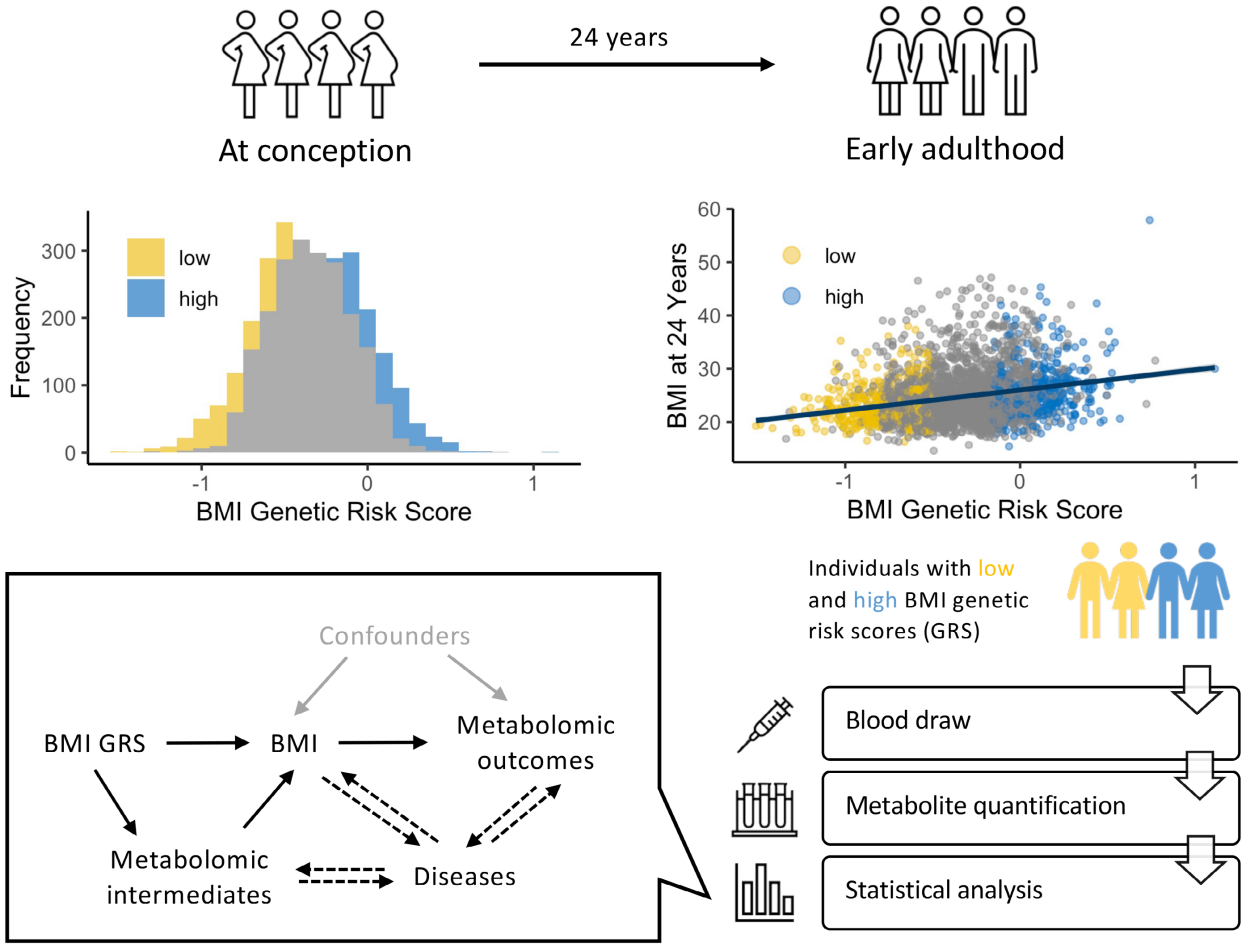
Study overview. This study involves the first‐generation offspring in the Avon Longitudinal Study of Parents and Children (ALSPAC) multigenerational cohort, in which 14,541 pregnant women, resident in the South West of England, were recruited in the 1990s. First, we constructed a genetic risk score (GRS) for BMI for all first‐generation offspring. Under the recall‐by‐genotype study design, we recalled the plasma samples (collected at the age‐24‐years clinic) of individuals with a low‐ (yellow) or high‐ (blue) BMI GRS for further analysis. Then metabolites in those plasma samples were quantified by Metabolon. Finally, we performed statistical analysis to compare the metabolite levels between the two BMI GRS groups. Our results are relevant to understanding the role of metabolites both as intermediates on the pathway to BMI and from BMI to disease. [Color figure can be viewed at wileyonlinelibrary.com]

In this study, metabolomics data were derived from plasma samples collected from a selection of participants of the Avon Longitudinal Study of Parents and Children (ALSPAC). An overview of the study design is shown in Figure 1.

### Study participants

ALSPAC is a prospective birth cohort of 14,541 pregnant women residing in the former region of Avon (UK) with expected dates of delivery from April 1, 1991, to December 31, 1992 (see Supporting Information Methods for a cohort summary) (8, 9, 10). A total of 13,988 children of the initial pregnancies who were alive at 1 year of age (Generation 1 [G1]) have been followed up with questionnaires and phenotypic assessments carried out during clinic visits. The study website contains details of all the data that are available through a fully searchable data dictionary and variable search tool (http://www.bristol.ac.uk/alspac/researchers/our‐data/). Our analysis included plasma samples and phenotype data from a subset of G1 participants and selected phenotype data for their parents. Ethical approval for the study was obtained from the ALSPAC Ethics and Law Committee and the local research ethics committees (http://www.bristol.ac.uk/alspac/researchers/research‐ethics/). Consent for biological samples was collected in accordance with the Human Tissue Act (2004). Informed consent for the use of data collected via questionnaires and clinics was obtained from participants following the recommendations of the ALSPAC Ethics and Law Committee at the time.

### Genotyping and sample selection

A subset of ALSPAC G1 participants (*N* = 8,953) were genotyped using the Illumina HumanHap550 quad chip and data imputed to the 1000 Genomes reference panel (Phase 1, Version 3; full details in Supporting Information Methods). A weighted GRS was calculated using PLINK (version 1.9) for all G1 participants with genetic data using a previously published set of 940 near‐independent genome‐wide significant BMI‐associated single‐nucleotide polymorphisms and their effect estimates (11). Following cross‐matching against those G1 participants with data and samples collected at the age‐24‐years clinic visit, those within the top and bottom 30% of the GRS distribution were selected for inclusion in the study. In what follows, these GRS‐derived groups will be referred to as the “high‐” and “low‐” BMI GRS groups, respectively. A total of 760 samples were sent for analysis, split equally between the high‐ and low‐BMI GRS groups. Further details of “GRS derivation” and “sample selection” are in the Supporting Information Methods.

### Derivation of metabolite data

Fasted blood samples were collected at the age‐24‐years clinic visit from all ALSPAC G1 individuals who provided informed consent (details in Supporting Information Methods). Plasma samples were assayed by Metabolon, Inc. (Durham, North Carolina) using ultrahigh performance liquid chromatography‐tandem mass spectroscopy (details in Supporting Information Methods). Metabolite screening identified 1,216 biochemicals, including 948 known (with the majority matched to purified standards) and 268 structurally unnamed biochemicals, as of December 2019 when data were generated (subsequent library updates are described in Supporting Information Methods). Original scale data normalized in terms of raw area counts (as supplied by Metabolon) were used.

### Phenotype data collection

In ALSPAC, regular clinic visits of subsets of G1 were carried out from 4 months to 24 years of age, including assessment of anthropometric measures, collection of biological samples, and completion of questionnaires. To validate the performance of the GRS, BMI (kilograms per meters squared) and weight (kilograms) data were extracted from all available time points. To further characterize the high‐ and low‐BMI GRS groups, total body fat mass (kilograms), total body lean mass (kilograms), waist‐hip ratio, and traditional measures of cardiometabolic health were extracted from the age‐24‐years clinic visit. To identify potential confounding due to (unmeasured) population structure, data were extracted for several phenotypic correlates of observed BMI to check for associations with GRS group and to evaluate their potential to act as confounders in the primary analysis. Selected variables from preexisting dietary preference data were extracted and used to proxy food intake in extended (post hoc) analyses. Details of phenotypic variables are in Supporting Information Methods.

### Statistical analysis

In all analyses, the low‐BMI GRS group was treated as the reference such that estimated effects represent the difference in the high‐BMI GRS group relative to the low‐BMI GRS group. All analyses were conducted in R Studio (12) using R version 4.0.2 (R Group) (13).

#### Metabolite processing

**FIGURE 2:**
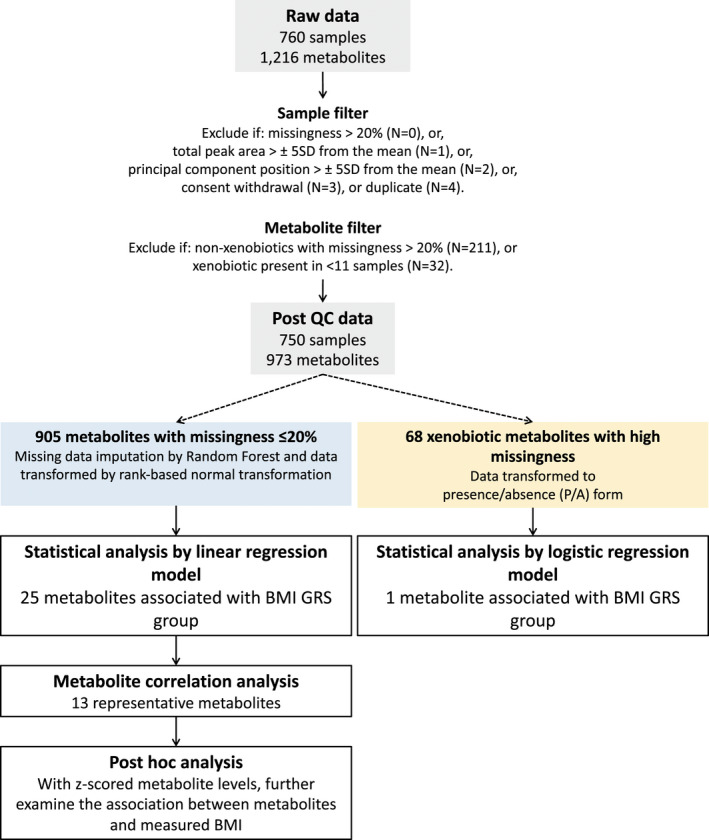
Overview of statistical analysis. “Raw data” is the original scale data normalized in terms of raw area counts (as supplied by Metabolon). Data were prepared for statistical analysis by first filtering samples and metabolites based on a series of quality metrics and then applying imputation and rescaling procedures as appropriate. GRS, genetic risk score; QC, quality control. [Color figure can be viewed at wileyonlinelibrary.com]

We processed the raw (original scale) data received from Metabolon (*N* = 760 samples) in preparation for statistical analysis using a prerelease version of *metaboprep* (14). Data were filtered based on a series of quality metrics. Nonxenobiotic metabolites with >20% missing values were excluded from the analysis. After exclusions, missing data were imputed using the random‐Forest‐based missForest R package and imputed data transformed using a rank‐based normal transformation. Xenobiotics (metabolites not produced by the human body) typically have a high level of missingness (or absence), which is both expected and biologically relevant given their (predominantly) exogenous origins. Therefore, xenobiotics with >20% missing were transformed to presence/absence (P/A) binary phenotypes. Xenobiotics present in <11 samples were excluded to ensure the robustness of downstream statistical analyses. A detailed description of this preanalysis processing is shown in Figure 2 and Supporting Information Methods.

#### Characterization of recall groups

Between‐group differences in our phenotype of interest, BMI, the previously described adiposity and metabolic health traits, and potential confounders (including technical covariates) were assessed. The normal distribution of continuous variables was checked by a Shapiro‐Wilk test. Between‐group differences of continuous variables were assessed primarily using a Student (two‐sample, two‐sided) *t* test assuming unequal variance; results from a two‐sample Wilcoxon (Mann‐Whitney) test are also presented for use in the case of non‐normally distributed traits (Shapiro‐Wilk W statistic < 0.90). A Fisher exact test for count data was applied to binary and categorical variables. Between‐group differences of BMI and weight were evaluated at different ages, ranging from 4 months to 24 years (when the samples were collected). Power calculations were performed using an online “Recall‐by‐Genotype Study Planner” application (7) as described in Supporting Information Methods.

#### Primary analysis: association of metabolites with recall group

To identify metabolite levels that differed between the low‐ and high‐BMI GRS groups, mean abundance was compared between groups using regression models. In Model 1, the postimputation rank‐based normal transformation metabolites were analyzed using linear regression (metabolite ~ BMI.GRS.group). The *R^2^* from the model was used to indicate the variance explained by GRS group. Log_2_ median fold change, calculated as the ratio of median abundance (untransformed and unimputed) in the high‐BMI GRS group divided by median abundance in the low‐BMI GRS group, was used to indicate relative effect sizes.

In Model 2, metabolites in the xenobiotic class with high levels of missingness and previously transformed to P/A traits were analyzed within a logistic regression framework (metabolite ~ BMI.GRS.group). The variance explained by the model was estimated using the “rsq” function in the R package “rsq” (15). A Benjamini‐Hochberg (BH) correction was applied to adjust the *p* values obtained from each of these analyses (Model 1 and Model 2) for multiple testing.

#### Extended analyses

Several post hoc analyses were carried out to further characterize the associations between BMI GRS group and the associated metabolites (BH *p* < 0.05) from Model 1. Analyses are described in brief here and a full description given in Supporting Information Methods.

Firstly, Model 1 was extended to a multivariate model in which any potential confounder that had previously been shown to be associated with GRS group was fitted as an independent fixed effect alongside GRS group. Furthermore, to assess the reproducibility of the observed associations in the absence of an independent replication data set, we performed a two‐step iterative resampling procedure based on that previously described in the context of genome‐wide association studies (16).

Secondly, a hierarchical clustering approach was applied to the subset of associated metabolites to identify redundancy in the data (i.e., in which associated metabolites were highly correlated and likely representing the same biological signal). A reduced set of “representative” metabolites was derived, forming the focus for the next steps.

Thirdly, linear regression analyses were conducted to evaluate the direct association between measured BMI (at the age‐24‐years visit) and the subset of BMI GRS group‐associated metabolites with BMI GRS group, sex, and age fitted as covariates (metabolite ~ BMI + BMI.GRS.group + sex + age). To investigate the consistency of the BMI effect across the two groups, the same model was also fitted with an interaction term (metabolite ~ BMI × BMI.GRS.group + sex + age) and within each BMI GRS group separately (metabolite ~ BMI + sex + age).

Finally, a series of hypothesis‐driven analyses was conducted to investigate the potential impact of dietary differences on our results. Previously collected data on food preference (at age 25 years) were used with a focus on specific food groups, as informed by the primary association analysis results. We tested for an association between the low‐ and high‐BMI GRS groups using a two‐sample Wilcoxon (Mann‐Whitney) test and for correlations between specific food groups and metabolites using linear regression.

## RESULTS

After filtering based on predefined quality metrics, the study sample consisted of samples from 750 individuals with abundance measures for 973 metabolites (905 continuous and 68 P/A traits). The phenotypic characteristics of all G1 individuals who attended the age‐24 clinic visit as compared with the study sample (after quality control) are presented in Table 1. Both recall groups were consistent with the overall cohort in terms of age and sex distribution, whereas adiposity traits showed expected differences.

**TABLE 1:** Characteristics of participants based on data collected at the age‐24‐years clinic

	All attending^a^ (*n* = 4,018)		Low‐BMI GRS (*n* = 373)		High‐BMI GRS (*n* = 377)		Between‐group difference^b^	
	*n*	Mean (SD)	*n*	Mean (SD)	*n*	Mean (SD)	OR/mean (95% CI)	*p* value
Sex, *n* (%)								
Male	1,504 (37.4)		148 (39.7)		150 (39.8)		1.00 (0.74 to 1.36)	1.00
Female	2,514 (62.6)		225 (60.3)		227 (60.2)			
Age								
Male	1,504	24.5 (0.80) y	148	24.6 (0.80) y	150	24.5 (0.73) y	−8.92 × 10^−4^ (−0.11 to 0.11)	0.99
Female	2,514	24.5 (0.82) y	225	24.4 (0.75) y	227	24.4 (0.81) y		
BMI								
Male	1,495	24.9 (4.44) kg/m^2^	148	23.8 (3.46) kg/m^2^	150	26.2 (4.66) kg/m^2^	2.78 (2.12 to 3.43)	3.79 × 10^−16^
Female	2,479	25.0 (5.42) kg/m^2^	222	23.1 (3.79) kg/m^2^	223	26.1 (5.62) kg/m^2^		
Weight								
Male	1,495	80.6 (15.3) kg	148	78.5 (12.6) kg	150	85.3 (17.1) kg	8.01 (5.72 to 10.3)	1.56 × 10^−11^
Female	2,481	68.8 (15.8) kg	223	63.4 (10.8) kg	223	72.1 (16.4) kg		
Total fat mass								
Male	1,459	20.6 (9.77) kg	146	18.6 (7.94) kg	144	23.4 (10.8) kg	5.67 (4.26 to 7.07)	1.17 × 10^−14^
Female	2,403	25.1 (11.1) kg	217	21.0 (7.79) kg	212	27.3 (10.9) kg		
Total lean mass								
Male	1,459	56.9 (7.55) kg	146	57.2 (7.05) kg	144	59.1 (8.20) kg	2.12 (0.60 to 3.63)	6.33 × 10^−3^
Female	2,403	41.2 (5.41) kg	217	40.0 (4.46) kg	212	42.1 (5.04) kg		
Waist‐hip ratio								
Male	1,493	0.85 (0.06)	148	0.84 (0.05)	150	0.85 (0.06)	0.02 (0.01 to 0.03)	1.07 × 10^−3^
Female	2,471	0.77 (0.06)	222	0.76 (0.05)	222	0.78 (0.06)		

Abbreviations: GRS, genetic risk score; OR, odds ratio.

^a^ Summary statistics based on all those who attended the age‐24‐years clinic.

^b^ Results from a Student two‐sample two‐sided *t* test to compare (sex‐combined) means in the high‐BMI GRS group with those in the low‐BMI group and expressed as an estimated difference in means. In the case of sex, a Fisher exact test was performed to test for a difference in the proportion of males vs. females in the two groups, and the results are presented as an OR.

### Characterization of recall groups

**FIGURE 3:**
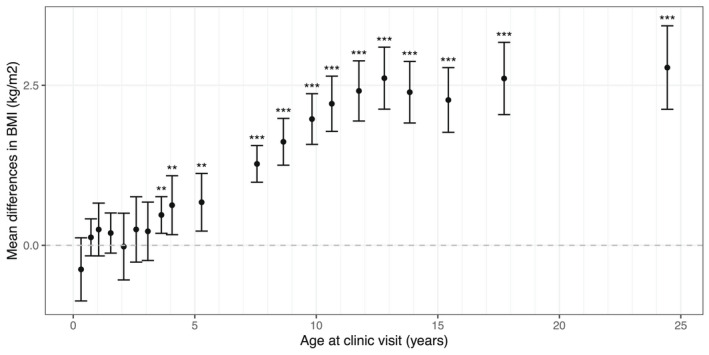
Mean differences in BMI between the high‐ and low‐BMI GRS groups. Error bars represent the 95% confidence interval of the mean difference in BMI. Sample size ranges from 108 (at age 31 months) to 743 (at age 24 years). Test results are given for a Student (two‐sample, two‐sided) *t* test. ***p < 0.001; **p < 0.01. For full results see Supporting Information Table S2

At the age‐24 clinic visit when the samples were collected, the mean (SD) BMI of individuals in the low‐BMI GRS group was 23.4 (3.7) kg/m^2^, falling within the “normal weight” range as defined by the World Health Organization (18.5 to <25 kg/m^2^). In contrast, the mean BMI of individuals in the high‐BMI GRS group, 26.1 (5.2) kg/m^2^, fell within the “pre‐obesity” range (25 to <30 kg/m^2^). Differences were also observed in weight, total fat mass, total lean mass, and waist‐hip ratio (Table 1). Several traditional measures of cardiometabolic health also showed differences across the groups, with the strongest association being observed in fasting insulin (Supporting Information Table S1). Temporal analyses showed that the between‐group differences in BMI emerged at about 4 years of age and then increased rapidly until participants reached around 13 years of age and somewhat more slowly thereafter (Figure 3 and Supporting Information Table S2); a similar pattern was observed in weight (Supporting Information Figure S1 and Table S2). BMI GRS group showed little association with most potential confounders tested and modest association with parental (mother’s and mother’s partner’s) social class (Supporting Information Table S3) but with no clear direction of effect across categories (Supporting Information Figure S2). Power calculations indicate that our RbG study is well‐powered to detect metabolite differences in which the variance in metabolite explained by BMI is similar to that for insulin (*R* ^2^ = 0.20, power = 94%). The minimum variance explained by BMI to achieve 80% power is *R* ^2^ = 0.126.

### Association of metabolites with recall group

**FIGURE 4:**
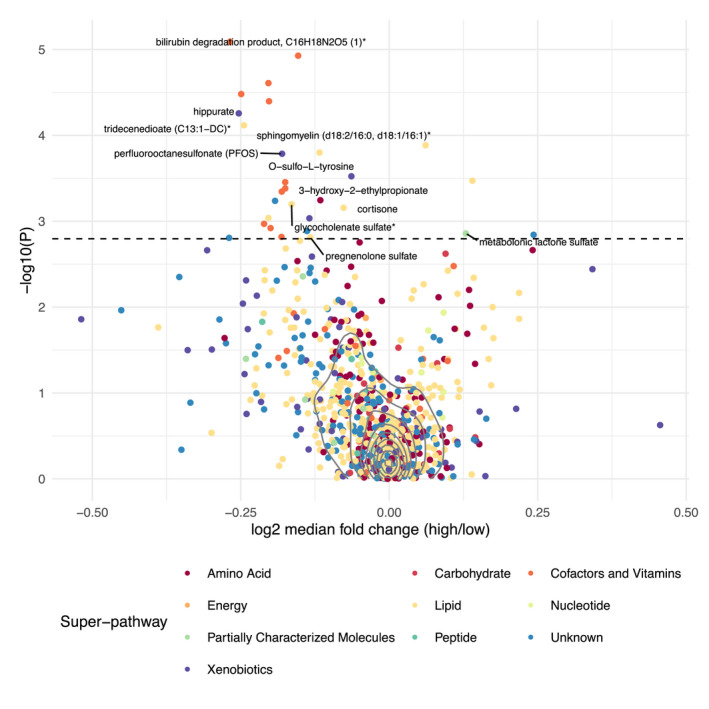
Volcano plot depicting the association between circulating metabolites and BMI genetic risk score (GRS) group. Points are colored by superpathway. Log_2_ median fold change calculated as the ratio of median abundance (untransformed and unimputed) in the high‐BMI GRS group divided by median abundance in the low‐BMI GRS group. *P* values used to derive −log_10_(*p*) are those from the linear regression analysis. All points above the dashed line have a Benjamini‐Hochberg adjusted *p* < 0.05. Solid gray lines indicate the density of points. A representative selection of metabolites of known identity are labeled. *Indicates a compound that has not been confirmed based on a standard. [Color figure can be viewed at wileyonlinelibrary.com]

Overall, we observed relatively small differences across a wide range of molecules with median log_2_ fold changes typically in the range −0.5 to 0.5 and a slight bias toward decreased abundances in the high‐BMI GRS group (Figure 4). Of the 905 metabolites tested in Model 1, 29 were associated with BMI GRS group (BH *p* < 0.05), 25 of which had annotations available from Metabolon (as of February 2020) (Table 2; see Supporting Information Table S4 for full results). Twenty‐five of twenty‐nine (86%) had lower mean abundance in the high‐BMI GRS group compared with the low‐BMI GRS group. The four metabolites that showed the greatest evidence for association with BMI GRS group were bilirubin and bilirubin degradation products from the “Hemoglobin and Porphyrin Metabolism” pathway. A total of 11 metabolites assigned to this pathway appeared in the list of associated metabolites, including biliverdin. GRS group allocation explained 2.6% of the variation in the abundance of the most strongly associated bilirubin degradation product. Four metabolites showed a positive association with the high‐BMI GRS group, including two forms of sphingomyelin and metabolonic lactone sulfate. For the 29 associated metabolites, within‐group distributions of metabolite levels were visualized using box and whisker plots with the original (unimputed abundance) data after mean centering and scaling as input (Supporting Information Figure S3).

**TABLE 2:** List of identified metabolites associated with BMI GRS group

Metabolite	Subpathway	*β* (95% CI)	BH *p* value	Cluster^a^
Bilirubin degradation product, C16H18N2O5 (1)^b^	Hemoglobin and porphyrin metabolism	−0.32 (−0.47 to −0.18)	0.005	1^c^
Bilirubin (Z,Z)	Hemoglobin and porphyrin metabolism	−0.32 (−0.46 to −0.18)	0.005	1
Bilirubin (E,Z or Z,E)^b^	Hemoglobin and porphyrin metabolism	−0.31 (−0.45 to −0.17)	0.007	1
Bilirubin degradation product, C16H18N2O5 (2)^b^	Hemoglobin and porphyrin metabolism	−0.30 (−0.44 to −0.16)	0.007	1
Biliverdin	Hemoglobin and porphyrin metabolism	−0.30 (−0.44 to −0.16)	0.007	1
Hippurate	Benzoate metabolism	−0.29 (−0.44 to −0.15)	0.008	2
Tridecenedioate (C13:1‐DC)^b^	Fatty acid, dicarboxylate	−0.29 (−0.43 to −0.15)	0.010	3^c^
Sphingomyelin (d18:2/16:0, d18:1/16:1)^b^	Sphingomyelins	0.28 (0.14 to 0.42)	0.015	4^c^
3‐Decenoylcarnitine	Fatty acid metabolism (acyl carnitine, monounsaturated)	−0.28 (−0.42 to −0.13)	0.015	3
Perfluorooctane sulfonate (PFOS)	Chemical	−0.27 (−0.42 to −0.13)	0.015	5
O‐Sulfo‐L‐tyrosine	Chemical	−0.26 (−0.41 to −0.12)	0.024	6
Sphingomyelin (d18:2/14:0, d18:1/14:1)^b^	Sphingomyelins	0.26 (0.12 to 0.40)	0.024	4
Bilirubin degradation product, C17H18N2O4 (2)^b^	Hemoglobin and porphyrin metabolism	−0.26 (−0.40 to −0.12)	0.024	1
Bilirubin degradation product, C17H18N2O4 (3)^b^	Hemoglobin and porphyrin metabolism	−0.26 (−0.40 to −0.12)	0.027	1
Bilirubin degradation product, C17H18N2O4 (1)^b^	Hemoglobin and porphyrin metabolism	−0.26 (−0.40 to −0.11)	0.027	1
3‐Hydroxy‐2‐ethylpropionate	Leucine, isoleucine, and valine metabolism	−0.25 (−0.39 to −0.11)	0.031	7
Glycocholenate sulfate^b^	Secondary bile acid metabolism	−0.25 (−0.39 to −0.11)	0.032	9
Cortisone	Corticosteroids	−0.25 (−0.39 to −0.11)	0.033	10
3‐Hydroxydecanoylcarnitine	Fatty acid metabolism (acyl carnitine, hydroxy)	−0.24 (−0.38 to −0.10)	0.040	3
Succinimide	Chemical	−0.24 (−0.38 to −0.10)	0.040	1
Bilirubin degradation product, C17H20N2O5 (1)^b^	Hemoglobin and porphyrin metabolism	−0.24 (−0.38 to −0.10)	0.044	1
Bilirubin degradation product, C17H20N2O5 (2)^b^	Hemoglobin and porphyrin metabolism	−0.24 (−0.38 to −0.10)	0.047	1
Metabolonic lactone sulfate	Partially characterized molecules	0.23 (0.09 to 0.38)	0.049	12
3‐Hydroxyoctanoylcarnitine (1)^b^	Hemoglobin and porphyrin metabolism	−0.23 (−0.37 to −0.09)	0.049	3
Pregnenolone sulfate	Pregnenolone steroids	−0.23 (−0.37 to −0.09)	0.049	14^c^

Note

Model fitted: metabolite ~ BMI.GRS.group (low‐BMI GRS group as reference group). Model run on rank‐based normal transformed metabolite data. *β* represents change in normalized SD units. Metabolites ordered by their BH adjusted *p* values from the lowest to the highest.

Abbreviations: BH, Benjamini‐Hochberg; GRS, genetic risk score.

^a^ Metabolite clusters assigned using an independent principal variables approach (clusters 8, 11, 13, and 15 contain a single unidentified metabolite each and are therefore not represented).

^b^ Indicates a compound that has not been confirmed based on a standard.

^c^ Representative metabolite for clusters consisting of more than one metabolite.

Of the 68 xenobiotic metabolites tested in Model 2, one metabolite, 2‐acetamidophenol sulfate, had evidence for association with BMI GRS group. This metabolite was present less often in the plasma samples of individuals from the high‐BMI GRS group (odds ratio = 0.59; 95% CI: 0.44‐0.79; BH *p* = 0.03). For full results of 68 metabolites from the logistic analysis, see Supporting Information Table S5.

### Extended analyses

Characterization of the GRS groups indicated some association with mother’s and mother’s partner’s social class. Therefore, in sensitivity analyses, these variables were fitted alongside GRS group for the 29 metabolites highlighted by the primary analysis. GRS group effect estimates from the multivariate model (Supporting Information Table S6) were similar to those from Model 1 (Pearson correlation, *r* = 0.99). In the two‐step iterative resampling analysis, 7 out of the top 10 associated metabolites surpassed the suggested threshold for robust association of 20 discovery and replication instances (Supporting Information Table S6). The number of successful discovery and replication instances was strongly correlated with the *p* value–based ranking from the primary association analysis.

Metabolite correlation analysis grouped the 29 metabolites output from Model 1 into 15 clusters, each with a representative metabolite (Table 2). The largest cluster consisted of 11 biochemicals, including two forms of bilirubin, seven bilirubin degradation products, biliverdin, and succinimide. There were 12 single metabolite clusters. Of the 15 representative metabolites, 12 were associated with measured BMI (*p* < 0.05) in a multivariate linear model with BMI and BMI GRS group fitted alongside age and sex, whereas 14 had effect estimates that were directionally concordant with their BMI GRS group association as derived in Model 1 (Supporting Information Table S7 and Figure S4).

**FIGURE 5:**
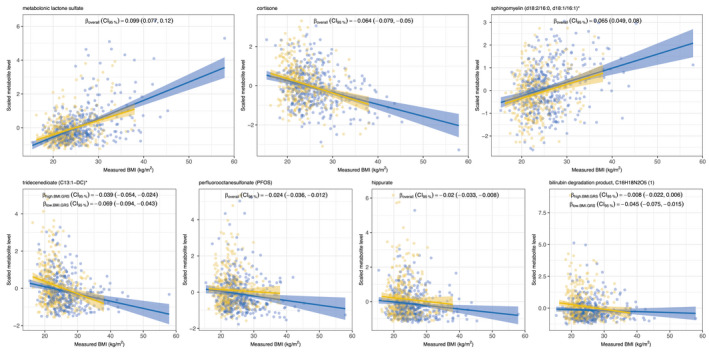
Relationship between selected BMI genetic risk score (GRS) group–associated metabolites and measured BMI. Based on measured BMI at age‐24‐years clinic visit. Yellow, low‐BMI GRS group; blue, high‐BMI GRS group. *β*_overall_ is the measured BMI effect (CI_95%_ = 95% CI), extracted from multivariate linear model fitted in all individuals (metabolite ~ BMI + BMI.GRS.group + sex + age). Where there was evidence that including an interaction term improved the fit of the model, the measured BMI effect (adjusted for age and sex) is given for each BMI GRS group separately (*β*_high.BMI.GRS_, *β*_low.BMI.GRS_). In the plots, solid lines denote the predicted univariate within GRS group relationship between BMI and metabolite with a 95% CI denoted by shading

Lower plasma levels of bilirubin degradation product (C16H18N2O5) (1), hippurate, perfluorooctanesulfonate (PFOS), tridecenedioate (C13:1‐DC), and cortisone as well as higher levels of sphingomyelin (d18:2/16:0, d18:1/16:1) and metabolonic lactone sulfate were associated with higher measured BMI and showed the same direction of association with measured BMI in both recall groups, concordant with the BMI GRS group association from the main analysis (Figure 5 and Supporting Information Table S7). Fitting an interaction term (measured BMI × BMI GRS group) in the model provided some evidence to support a difference in the measured BMI effect by BMI GRS group for bilirubin degradation product, C16H18N2O5 (1) (*p* = 0.048), and tridecenedioate (C13:1‐DC) (*p* = 0.046). There was less evidence to support an association of measured BMI with levels of 3‐hydroxy‐2‐ethylpropionate, pregnenolone sulfate, O‐sulfo‐L‐tyrosine, and glycocholenate sulfate (Supporting Information Figure S5 and Table S7).

Given the presence of potentially diet‐related molecules in the list of associated metabolites, a post hoc analysis of dietary data was performed. We focused on preference for fruits and vegetables because of their proposed relationship with hippurate and on fish preference because of the potential accumulation of PFOS in fish and crustaceans. There was a weak association (*p* = 0.04) between fish preference and BMI GRS group, with those in the high‐BMI GRS group showing a lower preference (Supporting Information Table S8A). Both fruit and vegetable preferences were associated with hippurate levels, including after adjustment for sex and BMI GRS group (Supporting Information Table S8B). Fish preference was associated with PFOS levels, but this association was attenuated on adjustment for sex and BMI GRS group (Supporting Information Table S8B).

## DISCUSSION

In this study, we characterized the metabolic profile associated with low versus high genetic liability for higher BMI using an RbG framework. The mean difference in BMI between BMI GRS groups increased from early childhood, reaching a maximum of 2.8 kg/m^2^ (95% CI: 2.1‐3.4 kg/m^2^) at time of sampling, when individuals were on average 24.5 years of age, reflecting differences in the ability of the GRS to capture variation in BMI at different ages, as shown previously (17). We identified 29 metabolites associated with BMI GRS group allocation. Most associated metabolites were seen at lower levels in the high‐BMI GRS group, with the largest effects seen for bilirubin, hippurate, and tridecenedioate. Two sphingomyelin metabolites were seen at increased abundance in the high‐BMI GRS group. The potential relevance of a selection of these metabolites to health and disease is explored in Supporting Information Table S9.

Conventionally, MR and RbG approaches attempt to isolate the causal contribution of modifiable exposures to chosen outcomes. However, when the outcome is also a biological intermediate that may itself be directly proxied by (elements of) the genetic predictor used to capture variance in the exposure, the assumptions underpinning causal inference no longer hold. Seven scenarios, including both “real” GRS‐to‐trait associations and associations that represent potential artifacts, may underpin observed associations between a GRS for disease and potential biomarkers (18). The scenarios, all of which could equally apply to the present study, include causal effects, reverse causality, associations due to biases or horizontal pleiotropy, and noncausal associations. Whereas others have concluded that the majority of metabolic perturbations seen in obesity are a response to increased adiposity itself (19), our results and those of Hsu et al. (20) suggest that differences in metabolism could also contribute to weight gain. Although both sets of metabolic pathways, cause and effect, may be informative with respect to predicting the risk of developing obesity‐associated comorbidities, only the former is likely to be of therapeutic relevance for the prevention of obesity. It is within this context that we go on to discuss our findings in more detail.

Bilirubin showed the strongest association with BMI GRS group allocation and, together with its degradation products, formed the largest cluster of associated metabolites. Bilirubin, which presents mostly as unconjugated (indirect) bilirubin in the body, is a key component of the heme catabolic pathway and was found in lower abundance in the high‐BMI GRS group. Circulating bilirubin has previously been found to be inversely associated with adiposity (19, 21, 22) and cardiovascular diseases (23, 24, 25). Genetic studies of bilirubin‐associated variants, including those in the *UGT1A1* gene that encodes a liver enzyme that converts unconjugated bilirubin into conjugated (direct) bilirubin, have been conducted to investigate the relevance of this molecule to health and disease. Although some connections have been made between bilirubin levels and type 2 diabetes (26) and hepatic damage (27) for example, MR studies have typically failed to support a causal role for the metabolite (25, 28). Recent experimental work supports an active role for bilirubin in improving cardiorenal and metabolic dysfunction potentially through activating nuclear receptors for burning fat (29) and reducing inflammation in adipose tissues (30).

Elevated levels of branched‐chain amino acids (BCAAs; leucine, isoleucine, and valine) and their tissue metabolites have been consistently detected in individuals with obesity (6). In this study, we saw little evidence for associations between BCAAs and BMI GRS group (Model 1 *β*s range from 0.015‐0.073 with unadjusted *p* values from 0.32‐0.84). Although this may seem at odds with previously identified correlations between BCAAs and adiposity, it is not totally unexpected given the level of inconsistency in observational and MR evidence (31, 32). For instance, a bidirectional MR study provided evidence for a causal effect of valine on BMI (20), although these results appeared to be instrument dependent, pointing to heterogeneity in the underlying biology. BCAA levels may also be influenced by dietary intake (33), and a link has been proposed between the obesity‐related rise in circulating BCAA levels and a decline in their catabolism in adipose tissue (34), with further evidence suggesting that this could be tissue specific (35). Moreover, 3‐hydroxy‐2‐ethylproprionate (a product of isoleucine catabolism) was observed to associate with BMI GRS group but not with measured BMI. One potential explanation for this is the differences in lean mass between groups given previously reported associations of 3‐hydroxy‐2‐ethylproprionate with muscle cross‐sectional area (i.e., with body composition) (36). However, we are not well‐powered to investigate this hypothesis within the current study.

We observed associations between BMI GRS group and the levels of potentially diet‐related metabolites, including hippurate and PFOS. Hippurate is a glycine conjugate of benzoic acid, of which the benzoic acid component is derived mainly via microbial and mammalian co‐metabolism of large polyphenolic molecules contained in, for example, fruits and vegetables (37). We observed lower levels of hippurate in individuals in the high‐BMI GRS group, concordant with its previous identified associations with visceral body fat mass (22, 38). Previous literature combining data on diet intake, visceral fat mass, and gut microbial profiling suggests that the association of circulating (and urine‐excreted) levels of hippurate with adiposity and related health outcomes (37) is likely to be the result of interactions between diet, microbiome composition, and adipose tissue function (22, 39). Post hoc analyses here also suggest that plasma hippurate levels are positively associated with fruit and vegetable intake. Individuals in the high‐BMI GRS group also had lower plasma levels of PFOS, an anthropogenic organic pollutant with chemical and thermal stability. PFOS has been detected in drinking water and the diet (especially in fish and crustaceans) worldwide and has a global toxic effect on human health. Although we see some evidence for greater preference for fish in the low‐BMI GRS group, sex also seems to be an important factor associated with PFOS levels. Several xenobiotics that showed between‐group differences (albeit not meeting our stringent threshold for association) may also be biomarkers of food consumption (e.g., acesulfame, betonicine, and theanine).

The associations observed between BMI GRS group and these metabolites suggest that at least some of the genetic predisposition to increased BMI may be conveyed either via dietary choices or through differences in nutrient metabolism. Many genes associated with high BMI appear to be highly expressed in the central nervous system (40) (e.g., through appetite regulation), which could be evidence that genetic susceptibility to obesity is partly attributable to appetitive phenotypes (41). However, behavioral traits such as these are known to be particularly at risk from bias even in an MR (and likely RbG) setting (42), in which population stratification (43) or complex genetic effects (e.g., dynastic effects (44)) that are not accounted for can be problematic. In this study, the weak correlation between GRS group and parental social class suggests some residual confounding (owing to population stratification) may be present. However, given the consistency in the effect estimates after adjusting for parental social class, we believe the effects of any such confounding on our results to be small.

There are a number of limitations to the current study design that could usefully be addressed in future work. Here we evaluated the impact of a genetic predisposition to a higher BMI on metabolite levels at a single point in time (early adulthood). This work could usefully be extended with longitudinal data to consider the extent to which these effects are consistent through the life course. The lack of gut microbiome metadata here prevents further exploration into the impact of a higher genetic predisposition to having overweight or obesity on the regulation of metabolite catabolism through nonhost factors. Finally, as is typical for untargeted analyses, here we used relative abundance (peak area) data rather than exact metabolite concentrations. In order both to validate our results and to consider their clinical relevance, targeted studies using verified standards are needed.

In conclusion, we used an innovative RbG study design to identify metabolites for which relative abundance varies with a genetic predisposition to increased BMI. These differences may reflect gene‐derived perturbations to biological pathways relevant to weight gain or they may be consequences of higher BMI itself. To answer this question, results from different approaches with unrelated sources of bias, including challenge and/or intervention studies, need to be integrated. In doing so, we can begin to understand the role of different metabolic pathways in weight gain and related morbidity and partition metabolites according to their relationship with increased adiposity.**O**

## CONFLICT OF INTEREST

The authors declared no conflict of interest.

## AUTHOR CONTRIBUTIONS

Conceptualization, Nicholas J. Timpson; methodology, Si Fang, David A. Hughes, Kaitlin H. Wade, Nicholas J. Timpson, and Laura J. Corbin; data acquisition: Sophie Fitzgibbon and Vikki Yip; formal analysis, Si Fang, Laura J. Corbin, Kaitlin H. Wade, Sophie Fitzgibbon, and Vikki Yip; writing: original draft, Laura J. Corbin and Si Fang; writing: review and editing, Nicholas J. Timpson, David A. Hughes, and Kaitlin H. Wade; supervision, Nicholas J. Timpson and Laura J. Corbin; funding acquisition, Nicholas J. Timpson. This publication is the work of the authors, and Laura J. Corbin and Nicholas J. Timpson will serve as guarantors for the contents of this paper.

## Supporting information

Fig S3

Table S1‐2 & S4‐S8

Supplementary Material

## Data Availability

The ALSPAC data used here are available on request to the ALSPAC executive committee (alspac-exec@bristol.ac.uk). The ALSPAC data management plan (available here: http://www.bristol.ac.uk/alspac/researchers/data‐access/) describes in detail the policy regarding data sharing, which is through a system of managed open access. Analysis code is available in GitHub from: https://github.com/lauracorbin/metabolomics_of_bmi/tree/v1.0 (DOI: 10.5281/zenodo.6451274).
