# Supplementary figures and images for: A multivariant recall‐by‐genotype study of the metabolomic signature of BMI
Source: Obesity (Silver Spring). 2022 May 22;30(6):1298–310. doi: 10.1002/oby.23441 (PMC9324973; doi:10.1002/oby.23441)

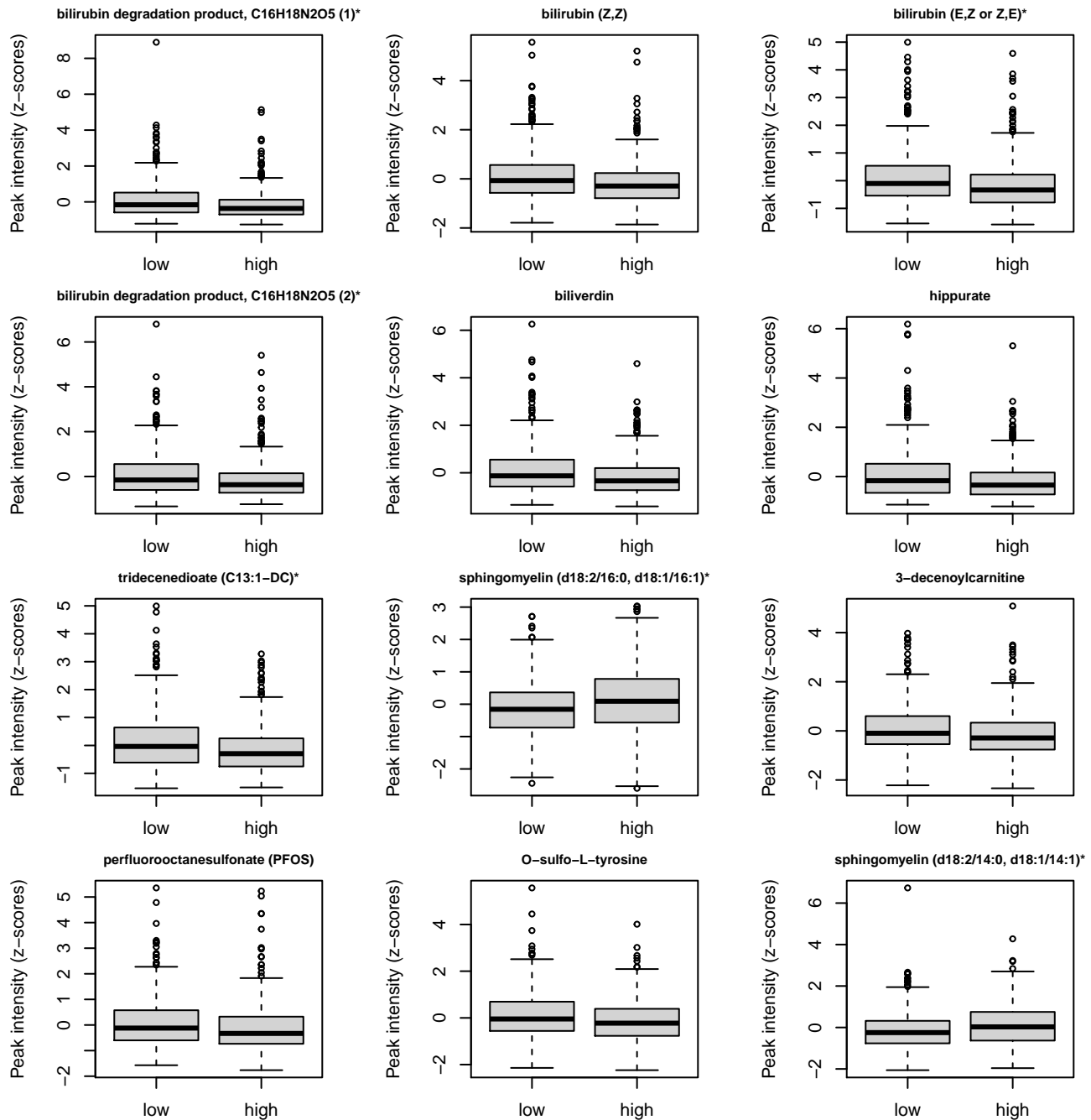

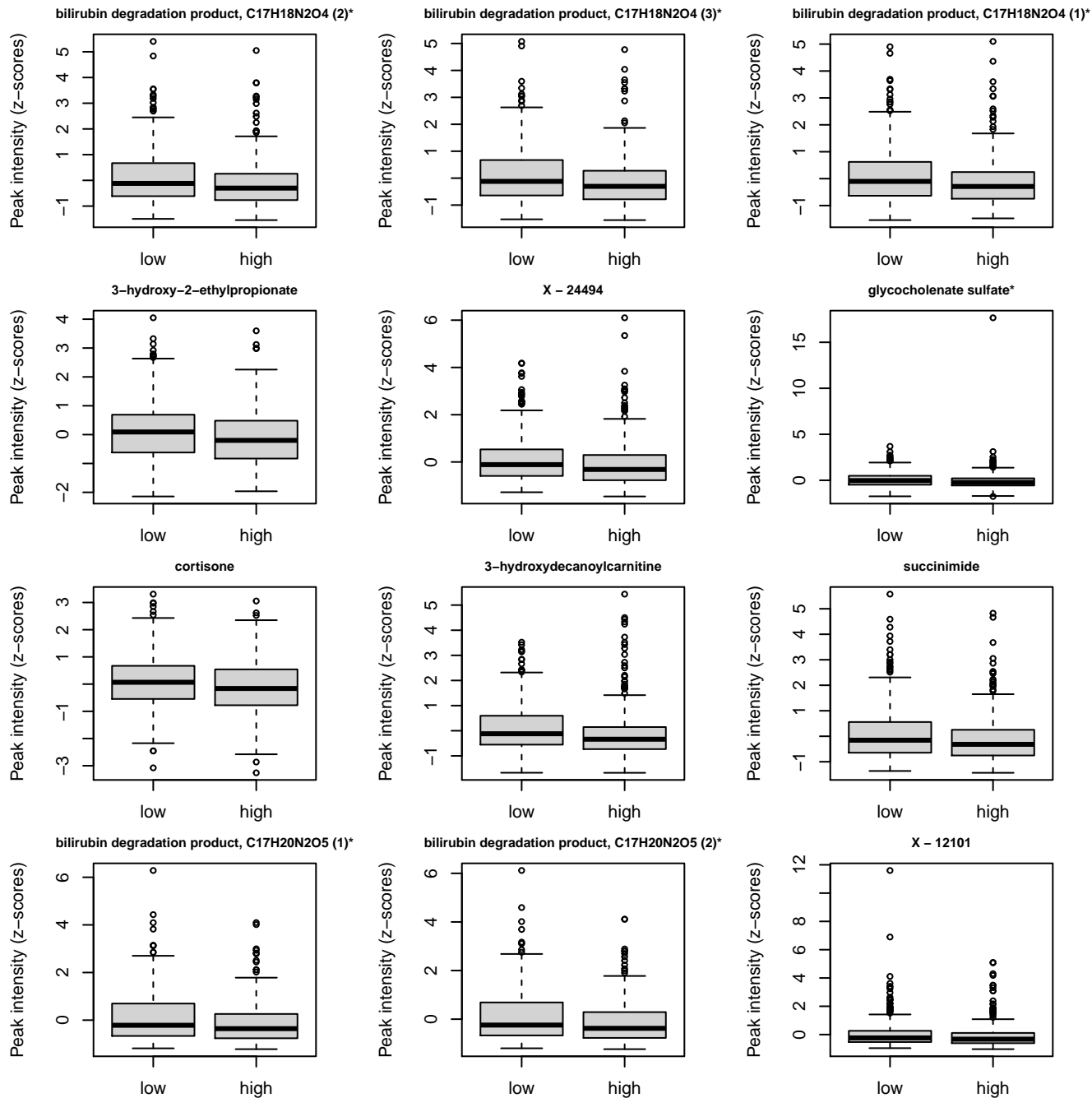

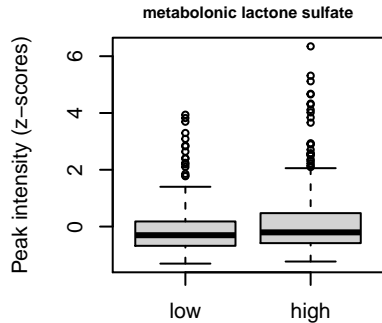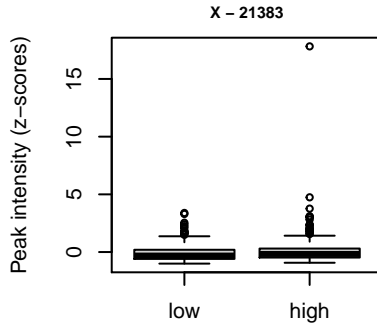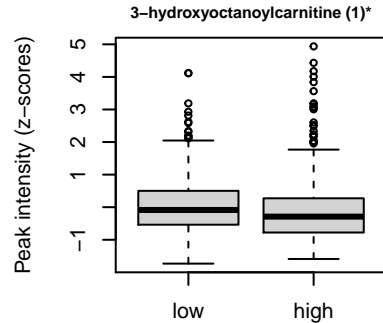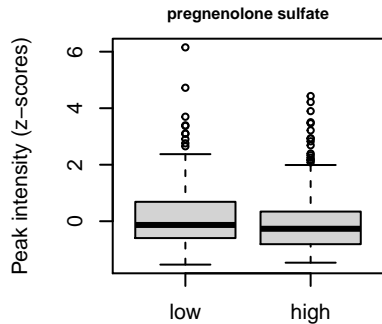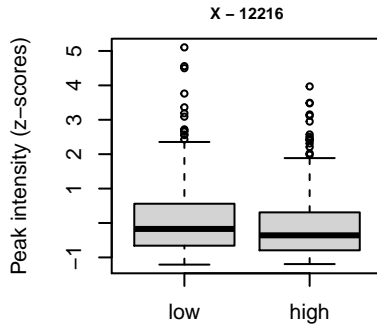

Supplement: Supplementary file 1 — Fig S3 [file OBY-30-1298-s003.pdf]
