# Supplementary material for: A multivariant recall‐by‐genotype study of the metabolomic signature of BMI
Source: Obesity (Silver Spring). 2022 May 22;30(6):1298–310. doi: 10.1002/oby.23441 (PMC9324973; doi:10.1002/oby.23441)
Supplement: Supplementary file 3 — Supplementary Material [file OBY-30-1298-s002.pdf]

Supporting Information for:

A multi-variant recall-by-genotype study of the metabolomic signatures of body mass index

Fang *et al*.

Table of Contents

Supplementary Methods ***4***

Avon Longitudinal Study of Parents and Children (ALSPAC): Cohort summary **4**

Description of study numbers 4

Data collection 4

Ethical approvals 5

Genotyping 5

Genetic risk score derivation **6**

Sample selection **6**

Sample collection **7**

Derivation of metabolite data by Metabolon, Inc. **7**

Sample preparation 7

Quality assurance / Quality control 7

Ultrahigh Performance Liquid Chromatography-Tandem Mass Spectroscopy (UPLC-MS/MS) 7

Data extraction and compound identification 8

Metabolite quantification and data normalization 8

Metabolite library updates 8

Definitions of variables **9**

Anthropometric traits 9

Traditional measures of cardiometabolic health 9

Potential confounders 10

Dietary information 11

Metabolite data pre-analysis processing **11**

Metabolite data processing pipeline 11

Selection of features for principal component analysis 12

Power calculations **12**

Extended analyses **13**

Extension of primary association analyses 13

Metabolite correlation analysis 13

Association of metabolites with measured BMI 14

Exploration of dietary factors 14

Supplementary Tables ***15***

Table S1. Between-group differences in traditional measures of cardiometabolic health measured at the 24 years of age clinic visit (in Excel file) **15**

Table S2. Between-group differences in BMI and weight from 4months to 24 years of age (in Excel file) **15**

Table S3. Association of potential confounders of primary analysis with BMI genetic risk score group **15**

Table S4. Results of linear regression for 905 metabolites (in Excel file) **16**

Table S5. Results of logistic regression for 68 metabolites (in Excel file) **16**

Table S6. Results of linear regression adjusting for maternal and paternal social class and two-step iterative resampling analysis for 29 metabolites associated with BMI genetic risk score group in the primary analysis (in Excel file) **16**

Table S7. Results of linear regression of metabolite levels on observed BMI for 29 associated metabolites (in Excel file) **16**

Table S8A. Between-group differences in food preference at 25 years of age (in Excel file) 16
Table S8B. Results of linear regression of metabolite levels on food preference 16
Table S9. Literature summary by metabolite 17
Supplementary Figures 22
Supplementary Figure S1. Mean differences in weight between the high and low BMI genetic risk score groups. 22
Supplementary Figure S2. Distribution across social class categories by BMI genetic risk score group 23
Supplementary Figure S3. Distribution of z-scored levels of BMI genetic risk score group associated metabolites by group. 24
Supplementary Figure S4. Comparison of BMI genetic risk score group effects estimated from Model 1 to BMI effect estimates based on measured BMI 24
Supplementary Figure S5. Relationship between selected BMI genetic risk score group associated metabolites and measured BMI. 25
Supplementary References 26

Supplementary Methods

Avon Longitudinal Study of Parents and Children (ALSPAC): Cohort summary

Description of study numbers

Pregnant women resident in Avon, UK with expected dates of delivery 1st April 1991 to 31st December 1992 were invited to take part in the study. The initial number of pregnancies enrolled is 14,541 (for these at least one questionnaire has been returned or a "Children in Focus" clinic had been attended by 19/07/99). Of these initial pregnancies, there was a total of 14,676 foetuses, resulting in 14,062 live births and 13,988 children who were alive at 1 year of age.

When the oldest children were approximately 7 years of age, an attempt was made to bolster the initial sample with eligible cases who had failed to join the study originally. As a result, when considering variables collected from the age of seven onwards (and potentially abstracted from obstetric notes) there are data available for more than the 14,541 pregnancies mentioned above. The number of **new pregnancies** not in the initial sample (known as Phase I enrolment) that are currently represented on the built files and reflecting enrolment status at the age of 24 is 913 (456, 262 and 195 recruited during Phases II, III and IV respectively), resulting in an additional 913 children being enrolled. The phases of enrolment are described in more detail in the cohort profile paper and its update (see footnote 4 below). The total sample size for analyses using any data collected after the age of seven is therefore 15,454 pregnancies, resulting in 15,589 foetuses. Of these 14,901 were **alive at 1 year of age**.

A 10% sample of the ALSPAC cohort, known as the **Children in Focus (CiF) group**, attended clinics at the University of Bristol at various time intervals between 4 to 61 months of age. The CiF group were chosen at random from the last 6 months of ALSPAC births (1432 families attended at least one clinic). Excluded were those mothers who had moved out of the area or were lost to follow-up, and those partaking in another study of infant development in Avon.

Data collection

Study data were collected and managed using REDCap electronic data capture tools hosted at the University of Bristol (1, 2). REDCap (Research Electronic Data Capture) is a secure, web-based software platform designed to support data capture for research studies, providing 1) an intuitive interface for validated data capture; 2) audit trails for tracking data manipulation and export procedures; 3) automated export procedures for seamless data downloads to common statistical packages; and 4) procedures for data integration and interoperability with external sources.

Please note that the study website contains details of all the data that is available through a fully searchable data dictionary and variable search tool (3).

Ethical approvals

Ethical approval for the study was obtained from the ALSPAC Ethics and Law Committee and the Local Research Ethics Committees (4). Consent for biological samples has been collected in accordance with the Human Tissue Act (2004). Informed consent for the use of data collected via questionnaires and clinics was obtained from participants following the recommendations of the ALSPAC Ethics and Law Committee at the time.

Genotyping

ALSPAC children were genotyped using the Illumina HumanHap550 quad chip genotyping platforms by 23andme subcontracting the Wellcome Trust Sanger Institute, Cambridge, UK and the Laboratory Corporation of America, Burlington, NC, US. The resulting raw genome-wide data were subjected to standard quality control methods. Individuals were excluded on the basis of gender mismatches; minimal or excessive heterozygosity; disproportionate levels of individual missingness (>3%) and insufficient sample replication (IBD < 0.8). Population stratification was assessed by multidimensional scaling analysis and compared with Hapmap II (release 22) European descent (CEU), Han Chinese, Japanese and Yoruba reference populations; all individuals with non-European ancestry were removed. SNPs with a minor allele frequency of < 1%, a call rate of < 95% or evidence for violations of Hardy-Weinberg equilibrium (P < 5E-7) were removed. Cryptic relatedness was measured as proportion of identity by descent (IBD > 0.1). Related subjects that passed all other quality control thresholds were retained during subsequent phasing and imputation. 9,115 subjects and 500,527 SNPs passed these quality control filters.

ALSPAC mothers were genotyped using the Illumina human660W-quad array at Centre National de Génotypage (CNG) and genotypes were called with Illumina GenomeStudio. PLINK (v1.07) was used to carry out quality control measures on an initial set of 10,015 subjects and 557,124 directly genotyped SNPs. SNPs were removed if they displayed more than 5% missingness or a Hardy-Weinberg equilibrium P value of less than 1.0e-06. Additionally, SNPs with a minor allele frequency of less than 1% were removed. Samples were excluded if they displayed more than 5% missingness, had indeterminate X chromosome heterozygosity or extreme autosomal heterozygosity. Samples showing evidence of population stratification were identified by multidimensional scaling of genome-wide identity by state pairwise distances using the four HapMap populations as a reference, and then excluded. Cryptic relatedness was assessed using a IBD estimate of more than 0.125 which is expected to correspond to roughly 12.5% alleles shared IBD or a relatedness at the first cousin level. Related subjects that passed all other quality control thresholds were retained during subsequent phasing and imputation. 9,048 subjects and 526,688 SNPs passed these quality control filters.

We combined 477,482 SNP genotypes in common between the sample of mothers and sample of children. We removed SNPs with genotype missingness above 1% due to poor quality (11,396 SNPs removed) and removed a further 321 subjects due to potential ID mismatches. This resulted in a dataset of 17,842 subjects containing 6,305 duos and 465,740 SNPs (112 were removed during liftover and 234 were out of HWE after combination). We estimated haplotypes using ShapeIT (v2.r644) which utilises relatedness during phasing. We obtained a phased version of the 1000 genomes reference panel (Phase 1, Version 3) from the Impute2 reference data repository (phased using ShapeIt v2.r644, haplotype release date Dec 2013). Imputation of the target data was performed using

Impute V2.2.2 against the reference panel (all polymorphic SNPs excluding singletons), using all 2186 reference haplotypes (including non-Europeans).

This gave 8,237 eligible children and 8,196 eligible mothers with available genotype data after exclusion of related subjects using cryptic relatedness measures described previously.

Genetic risk score derivation

Samples were selected for inclusion in the study based on a genetic risk score (GRS) for body mass index (BMI). We used publicly available summary statistics from the combined GIANT and UK Biobank meta-GWAS analysis of BMI (5) downloaded from: https://portals.broadinstitute.org/collaboration/giant/index.php/GIANT_consortium_data_files#BMI_and_Height_GIANT_and_UK_BioBank_Meta-analysis_Summary_Statistics (last accessed: October 2018), to calculate a weighted GRS. Specifically, we included 940 of the 941 SNPs listed in the paper as near-independent SNPs associated with BMI (at a revised genome-wide significance threshold of $P<1\times10^{-8}$) following an approximate conditional and joint multiple-SNP (COJO) analysis that considers LD between SNPs at a given locus (6). Within the set of 941 SNPs are 656 main associations and 285 secondary associations. The variant rs1000096 was the only SNP from the 941 not included in our GRS (due to a coding error).

Genetic risk scores were calculated for all ALSPAC G1 with genetic data (N=8,953). To generate the GRS, first, genotypes for the subset of 940 SNPs required were extracted from the full (imputed) ALSPAC dataset using QCTOOL v2. The .gen files produced for each chromosome were then concatenated to make a single .gen file with the "cat" command. The genotype data was then converted to dosage format, again, using QCTOOL v2 and manually reformatted to match the dosage format required for PLINK (including a swap of the reference allele). Finally, the PLINK --score function was used with the options: 'sum', 'double-dosage' and 'include-cnt' (7).

Sample selection

Individuals were ordered by their GRS and those within the top and bottom 30% of the distribution crossmatched against a list of individuals who both attended the age 24 years clinic visit and had a stored plasma sample available for analysis. This left N=738 individuals from the top 30% and N=806 from the bottom 30% in the eligible set. A scheme was then devised to select those with the most extreme GRS from the two groups (highest and lowest) whilst maintaining some degree of balance (in extremity) across the groups. First, individuals in the top set were assigned a rank, starting with the individual with the highest (most extreme) GRS. Conversely, individuals in the bottom set were assigned a rank starting with the individual with the lowest GRS. Based on these ranks and starting from rank 1, matched (on rank) pairs of individuals were selected for inclusion until 240 pairs had been selected. A further 138 individuals per set were then selected for inclusion, starting with those with the highest rank (i.e., the most extreme) in each set who had not yet been selected as part of a pair. This scheme resulted in an approximately balanced (by rank) sample of N=378 with the highest GRS in the top set and N=378 with the lowest GRS in the bottom set being selected for inclusion in the study. In addition, four duplicate samples were included (two per group). In total, 760 samples were sent to Metabolon for metabolomics analysis.

Sample collection

Blood samples were collected at the age 24 years clinic visit from all ALSPAC G1 individuals who provided informed consent. Participants were instructed to fast for a minimum of eight hours prior to their appointment and 90% complied (the remainder having consumed food within the preceding eight hours). During clinic appointments, blood was drawn into 10ml K2E (K2EDTA) tubes (VACUETTE®) that were centrifuged at 3500rpm for 10 minutes at 4–5°C. Plasma was then transferred to 200ul aliquots and stored at −80°C. Samples were processed and frozen within 90 minutes of collection, where possible. Selected samples were shipped on dry ice to Metabolon, Inc. for untargeted metabolomics analysis using established protocols (8, 9).

Derivation of metabolite data by Metabolon, Inc.

The methodological details provided herein are as supplied by Metabolon, Inc.

Sample preparation

Samples were prepared using the automated MicroLab STAR® system from Hamilton Company. Several recovery standards were added prior to the first step in the extraction process for QC purposes. Proteins were precipitated with methanol under vigorous shaking for 2 min (Glen Mills GenoGrinder 2000) followed by centrifugation. The resulting extract was divided into five fractions: two for analysis by two separate reverse phase (RP)/UPLC-MS/MS methods with positive ion mode electrospray ionization (ESI), one for analysis by RP/UPLC-MS/MS with negative ion mode ESI, one for analysis by HILIC/UPLC-MS/MS with negative ion mode ESI, and one for backup. Samples were placed briefly on a TurboVap® (Zymark) to remove the organic solvent. The sample extracts were stored overnight under nitrogen before preparation for analysis.

Quality assurance / Quality control

Three types of controls were used when analyzing the experimental samples: a pooled matrix sample generated by taking a small volume of each experimental sample (or alternatively, use of a pool of well-characterized human plasma); extracted water samples; and a cocktail of QC standards. Instrument variability was determined by calculating the median relative standard deviation (RSD) for the standards that were added to each sample prior to injection into the mass spectrometers. Overall process variability was determined by calculating the median RSD for all endogenous metabolites (i.e., non-instrument standards) present in 100% of the pooled matrix samples. Experimental samples were randomized across the platform run with QC samples spaced evenly among the injections.

Ultrahigh Performance Liquid Chromatography-Tandem Mass Spectroscopy (UPLC-MS/MS)

The Metabolon analysis consisted of four independent UPLC-MS/MS runs using different methods. All methods utilized a Waters ACQUITY ultra-performance liquid chromatography (UPLC) and a Thermo Scientific Q-Exactive high resolution/accurate mass spectrometer interfaced with a heated electrospray ionization (HESI-II) source and Orbitrap mass analyzer operated at 35,000 mass resolution. The sample extract was dried then reconstituted in solvents compatible to each of the four methods. Each reconstitution solvent contained a series of standards at fixed concentrations to ensure injection and chromatographic consistency. One aliquot was analyzed using acidic positive ion conditions, chromatographically optimized for more hydrophilic compounds. In this method, the extract

was gradient eluted from a C18 column (Waters UPLC BEH C18-2.1x100 mm, 1.7 µm) using water and methanol, containing 0.05% perfluoropentanoic acid (PFPA) and 0.1% formic acid (FA). Another aliquot was also analyzed using acidic positive ion conditions, however it was chromatographically optimized for more hydrophobic compounds. In this method, the extract was gradient eluted from the same afore mentioned C18 column using methanol, acetonitrile, water, 0.05% PFPA and 0.01% FA and was operated at an overall higher organic content. Another aliquot was analyzed using basic negative ion optimized conditions using a separate dedicated C18 column. The basic extracts were gradient eluted from the column using methanol and water, however with 6.5mM Ammonium Bicarbonate at pH 8. The fourth aliquot was analyzed via negative ionization following elution from a HILIC column (Waters UPLC BEH Amide 2.1x150 mm, 1.7 µm) using a gradient consisting of water and acetonitrile with 10mM Ammonium Formate, pH 10.8. The MS analysis alternated between MS and data-dependent MSn scans using dynamic exclusion. The scan range varied slighted between methods but covered 70-1000 m/z.

Data extraction and compound identification

Raw data was extracted, peak-identified and QC processed using Metabolon's hardware and software. Compounds were identified by comparison to library entries of purified standards or recurrent unknown entities. More than 3300 commercially available purified standard compounds have been acquired and registered into LIMS for analysis on all platforms for determination of their analytical characteristics. Additional mass spectral entries have been created for structurally unnamed biochemicals, which have been identified by virtue of their recurrent nature (both chromatographic and mass spectral).

Metabolite quantification and data normalization

Peaks were quantified using area-under-the-curve. A data normalization step was performed to correct variation resulting from instrument inter-day tuning differences.

Metabolite library updates

In December 2019, data for 948 known and 268 structurally unnamed biochemicals were returned by Metabolon. In February 2020, we sent a list of 'unknown' biochemicals that appeared in our list of associated metabolites to Metabolon requesting any new information from their current library. Metabolon returned a list of eight provisional identifications (not confirmed by standards): bilirubin degradation product, $C_{17}H_{20}N_2O_5$ (1), bilirubin degradation product, $C_{17}H_{20}N_2O_5$ (2), bilirubin degradation product, $C_{17}H_{18}N_2O_4$ (2), bilirubin degradation product, $C_{17}H_{18}N_2O_4$ (3), 3-hydroxyoctanoylcarnitine (1), bilirubin degradation product, $C_{16}H_{18}N_2O_5$ (2), bilirubin degradation product, $C_{16}H_{18}N_2O_5$ (1), and bilirubin degradation product, $C_{17}H_{18}N_2O_4$ (1).

In January 2022, Metabolon issued revised identifications for the following metabolites:

**Incorrect identification**	**Correct identification**
1-carboxyethylleucine	N-lactoyl leucine
1-carboxyethylisoleucine	N-lactoyl isoleucine
1-carboxyethylphenylalanine	N-lactoyl phenylalanine
1-carboxyethyltyrosine	N-lactoyl tyrosine
1-carboxyethylvaline	N-lactoyl valine
1-carboxyethylhistidine	N-lactoyl histidine

Definitions of variables

Anthropometric traits

Weight (kg) and BMI (kg/m^2): Weight (kg) and height (m) measures were assessed at all clinic visits. Standing height was measured to the nearest millimetre using a wall-mounted stadiometer. Weight was measured to the nearest 0.1kg using Tanita TBF-401A electronic body composition scales (or electronic bathroom scales, if the participant had a pacemaker). Body mass index (BMI) was calculated as [weight (kg)] / [height (m)^2]. In addition to the height and weight measures obtained at ALSPAC clinics, weight and BMI measures derived from other data sources (specifically mother-completed questionnaires and health visitor records) between the ages of four months and ten years were included in analyses (10).

Waist hip ratio: Waist and hip circumferences were measured to the nearest millimetre using a Seca 201 body tension tape and were repeated twice for accuracy. Waist hip ratio was calculated as [waist circumference (mm) / hip circumference (mm)].

Body composition phenotypes: Dual emission x-ray absorptiometry (DXA) was used to measure fat mass, muscle mass and bone density during the age 24 years clinic visit. Total body lean mass (g) and total body fat mass (g) were determined by full body DXA scans and transformed to kg prior to analysis.

Traditional measures of cardiometabolic health

Blood pressure: Seated blood pressure measurements taken during the age 24 years clinic were used. Blood pressure readings were taken using an Omron M6 upper arm blood pressure/pulse monitor. Participants were asked to sit and rest for two minutes prior to taking the seated blood pressure reading. Where possible, all blood pressure readings were taken using the participant's right arm.

Blood samples: Existing assay results available from fasting blood samples taken at the age 24 years clinic visit were used. Participants were eligible to have a blood sample taken provided they met the following criteria: 1) gave written consent; 2) did not have a clotting or bleeding disorder; 3) were not currently taking anti-coagulant drugs; and 4) not recently diagnosed as anaemic. Fasting blood samples were processed in EDTA blood tubes. Tubes were spun at 3500rmp for 10 minutes at 4-5°C (program 1 on the Thermo refrigerated centrifuge). After that, samples were split into aliquots and stored at -80°C within 90 minutes of collection. All subsequent assays for the measures included in this study were conducted at the Glasgow Cardiovascular Research Centre using kits manufactured by Roche Diagnostics GmbH, Sandhofer Strasse 116, D-68305 Mannheim. Measures included and their assays were:
Fasting insulin: Insulin levels were measured with a commercially available electrochemiluminescence immunoassay (ECLIA) kit (REF: 12017547 122).
Fasting glucose: Glucose was measured by the hexokinase method using the kit GLUC3 (Glucose HK) Cat. No. 04404483 190.
Triglycerides: Triglycerides were measured using enzymatic colorimetric tests with the kit TRIGL (Triglycerides) Cat. No. 20767107 322.

Fasting cholesterol: Cholesterol was measured using enzymatic colorimetric tests with the kit CHOL2 (Cholesterol gen.2) Cat. No. 03039773 190.
High density lipoprotein (HDL) cholesterol (HDL-C): HDL-C was measured using enzymatic colorimetric tests with the kit HDLC3 (HDL-Cholesterol plus 3rd generation) Cat. No. 04399803 190.
Low density lipoprotein (LDL) cholesterol (LDL-C): LDL-C was calculated using the Friedewald equation (LDL-C = total cholesterol minus (HDL-C + triglycerides/2.19)).
Very low-density lipoprotein (VLDL) cholesterol (VLDL-C): VLDL-C was calculated from triglyceride levels as VLDL = triglyceride / 2.19.
C-reactive protein (CRP): CRP was measured with a particle enhanced immunoturbidimetric assay using the kit CRPHS (Cardiac C-Reactive Protein (Latex) High Sensitive) Cat. No. 04628918 190.

Potential confounders

According to the theory of Mendelian randomization and analogous to the situation in a randomised controlled trial, creating groups based on a GRS for BMI (as opposed to using BMI itself), should ensure those groups do not differ in any other respect, meaning any downstream analyses should be free from confounding. However, in the presence of (unmeasured) population structure, associations can be induced between GRS (and therefore GRS group) and some of the traditional confounders of the relationship between BMI (as an exposure) and selected outcomes (11). Therefore, data were extracted for several phenotypic correlates of observed BMI to check for associations with GRS group and thus evaluate the potential for them to act as confounders in the primary analysis. Several phenotypes were selected measured in the G1 themselves (sex, age at the sample collection clinic (in weeks) and moderate to vigorous physical activity (MVPA) (minutes per day)), their mothers (parity, highest education, alcohol drinking status, smoking history, and social class) or their mother's partners (social class). Fasting status (binary) and time since last food/drink (in hours) were also evaluated as potential technical confounders.

Age (in weeks): Participant's age was recorded as their age at clinic visit. At different clinic visits, age was recorded in different units, including weeks, months and years. If age in weeks data were available, the age was used in the present study directly. Otherwise, age data were converted to weeks by multiplying age in months data by 4.34524.

Moderate to Vigorous Physical Activity (MVPA) (minutes per day): At the age 24 years clinic, participants were asked to wear an ActiGraph GT3X+ accelerometer device after the clinic visit for four consecutive days which were part of a "normal week" instead of doing anything unusual. An objective measurement of human activity was measured by the device. MVPA referred to activities with a counts per minute (cpm) of more than 2020. MVPA minutes used in the present study was defined as the average of MVPA minutes per day over all days the device was worn.

Parental phenotypes: Phenotypic data relating to the participant's mother (parity, smoking history, alcohol drinking status, highest education, and social class), and her partner's social class were collected via questionnaires completed by the mother during pregnancy. Parity is defined as the number of previous pregnancies resulting in either a livebirth or a stillbirth. Mother's smoking history is defined as the mother's answer to "Have you ever been a smoker?", being either "Yes" or "No". Mother's alcohol drinking status records the alcohol

consumption frequency of the mother before the current pregnancy with six categories ("never", "<1 glass per week", "1+ glasses per week", "1-2 glasses per day", "3-9 glasses per day", "10+ glasses per day"). Mother's highest education qualification is a categorical variable with five values ("CSE/none", "Vocational", "O level", "A level" and "Degree"). Mother's and partner's social classes are derived variables generated based on the type of industry and level of their occupation with seven different values.

Fasting status: Two variables, recorded during the age 24 years clinic visit, were used to assess fasting status. Participants were instructed to fast for a minimum of eight hours before attending the clinic during which blood sampling was undertaken. Therefore, during the clinic visit, participants were asked when they last ate or drank and two variables derived: (1) a continuous phenotype – time since last food/drink (in hours); a binary phenotype indicating whether food/drink had been consumed in the last 8 hours or not, i.e., fasting status.

Dietary information

Dietary preference data was collected as part of the *Life @ 25+* questionnaire that was sent to study participants when they were approximately 25 years of age. Participants were asked to rate how much they liked each one of 97 food and drink items using a scale between 1 (extremely dislike) and 9 (extremely like). If participants were unfamiliar with or had not tasted any of the foods, they could select a 'never tasted' option (coded as '0').

For this study, we derived three composite measures of intake by summing individual preferences (after centering and scaling) as follows:
Overall fish preference: Calculated as the sum of fried/battered fish, baked/steamed fish, prawns, salmon, shellfish, smoked salmon and tuna.
Overall vegetable preference: garlic, green olives, mushrooms, onions, tomatoes, chilli peppers, artichokes, asparagus, aubergines, avocados, black olives, broad beans, broccoli, brussels sprouts, cabbage, carrots and spinach.
Overall fruit preference: apples, bananas, cherries, dried fruit, lemons, oranges, pears and strawberries.

Metabolite data pre-analysis processing

Metabolite data processing pipeline

We processed the raw (original scale) data received from Metabolon (N=760 samples) in preparation for statistical analysis using an in-house pipeline developed in R (12) (a pre-release version of the R package 'metaboprep' currently available from: https://github.com/MRCIEU/metaboprep) (13). First, all metabolites designated (by Metabolon) as xenobiotics were temporarily removed from the dataset. The reason for this is that xenobiotics are metabolites not produced by the body, such as drug compounds, and therefore can have very high rates of missingness, while still being critically informative to a study. Next, we screened for particularly poorly performing samples and metabolites, defined as those with >80% missing data. Missingness was then re-assessed based on remaining samples and metabolites and a more stringent missingness criteria of >20% applied (i.e., samples and metabolites with >20% missing values removed). No samples were excluded based on missingness at either threshold, whilst 211 metabolites were removed (22 with >80% missing and a further 189 with >20% missing). Sample quality was

further assessed based on total peak area (TPA), calculated as the sum of all metabolite values measured in a sample, with a single sample whose TPA fell more than five standard deviations (SDs) from the mean being excluded. Finally, a principal component analysis (PCA) was conducted on the samples using a subset of approximately independent metabolites (see below for details) to identify potential outliers. Two samples positioned more than five SDs from the mean of the first and/or second principal components were considered outliers and excluded from subsequent analysis. Following these procedures, data for the xenobiotic metabolites were added back into the dataset. Further sample exclusions were applied based on consent withdrawals (N=3) and duplicate samples (N=4). In the case of the latter, samples with the least missing data from each pair were retained for analysis. After processing, 750 samples (377 from high BMI GRS group and 373 from low BMI GRS group) and 1005 metabolites remained.

Of the 1005 metabolites remaining after processing, 905 were (non-xenobiotic) metabolites with <20% missing data. In this dataset, missing data was imputed using a random-forest based method implemented in the missForest R package (14). Then, data for this set of metabolites were transformed by rank-based normal transformation (RNT) to ensure normality prior to statistical analysis. RNT was achieved using a modified version of the rntransform() function (from the GenABEL package) such that any tied values were randomly split (code is available from the moosefun git repository at: https://github.com/hughesevoanth/moosefun). Meanwhile, data for the 100 xenobiotic metabolites with >20% missing data were transformed to presence/absence (P/A) data, where 1 represents presence in a sample and 0 represents absence. Finally, xenobiotics present in less than 11 samples (N=32) were excluded from downstream analyses on the basis that any statistical analyses would not be robust.

Selection of features for principal component analysis

Prior to conducting the PCA to identify sample outliers, a subset of approximately independent features was selected as follows. Data were restricted to common metabolites such that only those that were (a) variable and (b) had at least 50 observations were included. A dendrogram was then constructed (stats package hclust() function, with method 'complete') based on a Spearman's rho distance matrix (1-|Spearman's rho|). A set of 'k' clusters (groups of similar metabolites) were identified based on a user-defined tree cut height 0.2 (equivalent to a Spearman's rho of 0.8), using the function cutree() from the stats package. For each 'k' cluster the metabolite with the least missingness was then tagged as the representative metabolite for that cluster. Data for the subset of representative metabolites then underwent imputation with missing values imputed to the median and finally data standardised (z-transformed) such that the mean equals zero and the standard deviation equals one for each metabolite. These data were then used as input to the PCA analysis conducted as part of the data processing pipeline described above.

Power calculations

An online 'Recall by Genotype Study Planner' application (15), version Beta 2.5 (available at: http://ieushiny.biocompute.org.uk/rbg-app/) was used to evaluate the power of our study using an analytical approach. Amongst the input parameters required is the R^2 between the

GRS and the exposure (here, BMI); we used a value of R^2=0.061, as calculated using data from all participants with genetic data and BMI measured at the age 24 years clinic (N=3076). In addition, an estimate of the R^2 between the exposure (BMI) and the outcome(s) (metabolites) is needed. We used existing blood assay measures to estimate a realistic range of R^2. Based on existing data from the age 24 years clinic, the R^2 between BMI and the range of blood measures tested were as follows: fasting glucose R^2=0.012 (N=3218); cholesterol R^2=0.020 (N=3218); LDL R^2=0.060 (N=3216); VLDL R^2=0.083 (N=3216); triglycerides R^2=0.071 (N=3217); and, fasting insulin R^2=0.201 (N=3218). Power was estimated based on the highest exposure-outcome R^2 value (R2=0.201) and the minimum R^2 value required to achieve 80% power calculated. The sample size was fixed at N=750 and the alpha at 0.05.

Extended analyses

Extension of primary association analyses

For the metabolites found to be associated with recall group in Model 1, extension analyses were carried out as follows.

Model 1 was extended to a multivariate model in which any potential confounder that had previously been shown to be associated with BMI GRS group was fitted as an independent fixed effect alongside BMI GRS group. In addition to extracting the model coefficients, the variance explained by each of the fixed effects in the model (designated 'VE' in results files) was estimated by Type II ANOVA ('Anova()' function from the 'car' package in R (16)).

To assess the reproducibility of the observed associations in the absence of an independent replication dataset we performed a two-step iterative resampling (TSIR) procedure based on that previously described in the context of genome-wide association studies (17). This analysis was conducted using the same data as was used for the '*Primary analysis - association of metabolites with recall group*'. The recall-by-genotype cohort (N=750) was randomly split into discovery (70%, N=525) and replication (30%, N=225) cohorts. Using the discovery cohort, mean metabolite abundance was compared between groups using regression Model 1 (as described in the main methods); metabolites were considered to be associated if they had p<0.002 (a threshold approximately equal to the Benjamini-Hochberg-corrected p-values used in the primary analysis). The same analysis was then conducted in the replication cohort with p<0.05 indicative of replication. This discovery-replication process was repeated 100 times and the number of times each metabolite was associated both in the discovery and replication cohorts recorded, giving a replication value between 0 and 100 where the higher the value, the more robust the association. It has been suggested that an exposure be designated as 'associated' if it is discovered/replicated at least 20 times (out of 100) (17).

Metabolite correlation analysis

We took the subset of associated metabolites output from Model 1 and used a hierarchical clustering approach to identify redundancy in the data (i.e., where associated metabolites were highly correlated and likely representing the same biological signal). This was done based on the original (unimputed abundance) data and using the iPVs R package (18) with a tree cut height of 0.75 (where the value is equal to a dissimilarity of 1 - Spearman's rho). As

well as assigning metabolites to clusters based on their similarity, this package uses principal variable analysis (PVA) to select the best representative metabolite of each cluster. The set of reduced 'representative' metabolites was used as the focus for the next steps.

Association of metabolites with measured BMI

A series of linear regression analyses were conducted to evaluate the direct association between measured BMI (at the age 24 years clinic) and the subset of BMI GRS group associated metabolites. Having established that the (associated) metabolite distributions were approximately normal, original (unimputed abundance) data was mean centred and scaled by the standard deviation (z-scored) and the overall association between measured BMI and metabolite level assessed with BMI GRS group, sex and age fitted as covariates in a multivariate linear model [metabolite ~ BMI + BMI.GRS.group + sex + age]. In order to investigate the consistency of the BMI effect across the two groups, the same model was also fitted with an interaction term [metabolite ~ BMI * BMI.GRS.group + sex + age] and within each BMI GRS group separately [metabolite ~ BMI + sex + age]. Robust standard errors generated with coeftest() from lmtest R package were reported because of heteroscedasticity. ANOVA tests were carried out to ascertain whether including the interaction effect improved the model fit. Variance explained by each fixed effect in the model was calculated as described previously.

Exploration of dietary factors

Composite measures of fish, vegetable and fruit preference (derived from food preference questionnaires conducted at age 25 years) were derived (as described above) and used as proxy measures for intake of these foods at the time of blood sampling (the age 24 years clinic). Two-sample Wilcoxon (Mann-Whitney) tests were conducted to test for between-group differences in these variables. Linear regression models were used to test for an association between specific food groups and metabolites [metabolite ~ diet.variable] and additionally adjusting for sex and BMI GRS group [metabolite ~ diet.variable + BMI.GRS.group + sex]. Metabolites were centred and scaled and, where appropriate, log transformed prior to regression modelling.

Supplementary Tables

Table S1. Between-group differences in traditional measures of cardiometabolic health measured at the 24 years of age clinic visit (in Excel file)

See separate Excel file.

Table S2. Between-group differences in BMI and weight from 4months to 24 years of age (in Excel file)

See separate Excel file.

Table S3. Association of potential confounders of primary analysis with BMI genetic risk score group

Phenotype category	Statistical test	Phenotype	Mean difference (95% CI)[a]	p-value
Continuous	Student's two-sample two-sided t-test	MVPA (minutes)	-1.02 (-10.1,8.02)	0.82
		Age at clinic (weeks)	-0.05 (-5.85,5.76)	0.99
		Time since last food at sampling (hours)	-0.21 (-0.70,0.28)	0.40
Categorical	Fisher's exact test for count data	Mother's highest education	na	0.15
		Mother's social class	na	0.08
		Mother's partner's social class	na	0.02
		Mother's parity	na	0.83
		Mother's alcohol drinking status (frequency)	na	0.29
Binary		Mother's smoking status	0.87 (0.63,1.19)	0.40
		Fasting status at sampling	0.69 (0.40, 1.20)	0.19

CI = confidence interval;

[a] expressed as the mean difference in the high BMI genetic risk score group as compared to the low BMI genetic risk score group as the reference.

Table S4. Results of linear regression for 905 metabolites (in Excel file)

See separate Excel file.

Table S5. Results of logistic regression for 68 metabolites (in Excel file)

See separate Excel file.

Table S6. Results of linear regression adjusting for maternal and paternal social class and two-step iterative resampling analysis for 29 metabolites associated with BMI genetic risk score group in the primary analysis (in Excel file)

See separate Excel file.

Table S7. Results of linear regression of metabolite levels on observed BMI for 29 associated metabolites (in Excel file)

See separate Excel file.

Table S8A. Between-group differences in food preference at 25 years of age (in Excel file)

See separate Excel file.

Table S8B. Results of linear regression of metabolite levels on food preference

See separate Excel file.

Table S9. Literature summary by metabolite

Metabolite name	Summary of findings	General information	Relevance to disease
**Bilirubin**	A total of 10 bilirubin related metabolites (including biliverdin) were found to be lower in individuals in the high BMI genetic risk score (GRS) group.	Bilirubin is a key component of the heme catabolic pathway, in which the heme present in hemoproteins from red blood cell catabolism are oxygenated (by heme oxygenase) to biliverdin, and then reduced by biliverdin reductase to bilirubin.	Bilirubin has been observed to have a protective role against inflammation, hepatic diseases, diabetes, metabolic syndrome and obesity (19–22). Mendelian randomization (MR) studies found that genetically predicted higher levels of bilirubin is associated with a lower risk of cardiovascular diseases (23–25) but higher risks of gallstone diseases (26) and colorectal cancer (27). However, MR results have yet to provide robust evidence of a causal contribution from bilirubin on type 2 diabetes (24, 28).
**Sphingomyelin**	Sphingomyelin (d18:2/16:0, d18:1/16:1) and sphingomyelin (d18:2/14:0, d18:1/14:1) showed higher levels in individuals from high BMI GRS group.	Sphingomyelins are the most abundant class of sphingolipids in lipoproteins with essential roles in both maintaining plasma membrane structures and cellular signalling (29). Sphingomyelin consist of multiple species, with sphingosine (d18:1) being the most prominent long-chain base and palmitic acid (16:0) as the most common fatty acid component (29, 30).	It has been long recognised that high levels of plasma sphingomyelin promote atherogenesis, and it is an independent risk factor for coronary artery disease (31, 32).

Metabolite name	Summary of findings	General information	Relevance to disease
**Hippurate**	We observed lower levels of hippurate in individuals in the high BMI GRS group and a corresponding negative association with measured BMI.	Hippurate, or hippuric acid, is a mammalian-microbial cometabolite synthesised by glycine conjugation of benzoic acid in the liver and kidney and from microbial metabolism of diet-derived polyphenols (33).	Higher levels of hippurate have previously been associated with higher fruit and whole grain intake, lower visceral fat mass and reduced risk of having metabolic syndrome features (34–36).
**PFOS**	We found individuals in the high BMI GRS group had lower plasma PFOS levels and observed a corresponding negative association with measured BMI.	PFOS is one of the many anthropogenic organic pollutant perfluoroalkyl and polyfluoroalkyl substances (PFASs) with chemical and thermal stability. They have been widely applied as industrial and commercial polymers and surfactants since 1950 (37). Despite most uses of PFOS having been phased out, banned or restricted under a number of UK, EU and international regulations, PFOS remains a widespread environmental contaminant (38). Studies published as recently as 2019 have confirmed the presence of PFOS in drinking water in multiple countries and areas across the globe (39) and the diet in Canada (40), Spain (41), the Netherlands (42) and Greece (43), especially in fish and crustaceans.	In humans, PFOS accumulates in multiple tissues and body fluids with enrichments in blood (44) and the liver (45). Molecular and animal studies have shown that PFOS has a global toxic effect on human health, including detrimental impacts on the liver, neuron system, reproductive system and immune system (46). In Japan, data has shown serum PFOS levels to vary by time, sex and geography (47).

**Metabolite name**	**Summary of findings**	**General information**	**Relevance to disease**
**Cortisone**	We observed lower levels of cortisone among individuals in the high BMI GRS group and this association was supported in the analysis of measured BMI.	Cortisone is an inert metabolite of cortisol, a steroid hormone released by the adrenal cortex. Cortisone regenerates cortisol, and its levels are regulated by reversible enzyme shuttle between two subtypes of 11β-Hydroxysteroid dehydrogenase (11β-HSD) (48).	There is a long history of research into cortisol, the precursor of cortisone, and obesity (49). Whilst a cross-sectional study and review of the literature published in 2013 concluded there was no strong relationship between systemic cortisol and obesity (50), more recent work has pointed to associations between both cortisone and cortisol and specific fat depots (36).
**O-sulfo-L-tyrosine**	The level of O-sulfo-L-tyrosine was found to be lower in individuals with higher BMI GRS, but it did not show strong association with measured BMI in both recall groups.	O-sulfo-L-tyrosine is a metabolite from the class of phenylalanine and derivatives.	Previous studies have identified an association between higher O-sulfo-L-tyrosine levels and reduced kidney function in chronic kidney disease (51, 52).
**3-hydroxy-2-ethylpropionate**	Low levels of 3-hydroxy-2-ethylpropionate were observed in the high BMI GRS group, but it did not show strong association with measured BMI in both recall groups.	3-hydroxy-2-ethylpropionate, or 2-ethylhydracrylate, is a metabolite involved in the metabolism of leucine, isoleucine and valine, the branched chain amino acids.	3-hydroxy-2-ethylpropionate has been found to be positively associated with muscle cross-sectional areas and reversely associated with the proinflammatory cytokines tumour necrosis factor-α (TNF-α) (53).

**Metabolite name**	Summary of findings	General information	Relevance to disease
**Glycocholenate sulfate***	Low levels of glycocholenate sulfate were observed in the high BMI GRS group, but it did not show strong association with measured BMI in both recall groups.	Glycocholenate sulfate is a circulating secondary bile acid.	Circulating glycocholenate sulfate level was found to be positively associated with incidence of atrial fibrillation (AF) risk in multiple studies (54, 55). Another study suggested it was inversely associated with high-grade glioma (56).
**Metabolonic lactone sulfate**	High levels of metabolonic lactone sulfate were observed in the high BMI GRS group, and this association was supported in the analysis of measured BMI in both recall groups.	Metabolonic lactone sulfate is a partially characterized molecule with currently unknown biological function.	In a recent study on Mexican and African Americans, plasma levels of metabolonic lactone sulfate were found to associated with anthropometric traits (including BMI and measures indicating central adiposity), and indicators of cardiometabolic health (e.g., insulin sensitivity, triglycerides, diastolic blood pressure) (57). In addition, it was found to be inversely associated with the echogenicity of the intima-media complex of the common carotid artery, which was associated with increased risks of both myocardial infarction and stroke (58).

Metabolite name	Summary of findings	General information	Relevance to disease
**3-hydroxydecanoylcarnitine**	Low levels of this metabolite were observed in the high BMI GRS group, and this association was supported in the analysis of measured BMI in both recall groups.	3-hydroxydecanoylcarnitine is a medium chain acylcarnitine, a class of metabolites which are involved in the transportation of organic acids and fatty acids for energy generation in the mitochondria.	Urinary levels of 3-hydroxydecanoylcarnitine were observed to be correlated with chronic environmental metal and benzene exposure (59).
**Pregnenolone sulfate**	Low levels of pregnenolone sulfate were observed in the high BMI GRS group, and it showed reverse association with measured BMI in low BMI GRS recall group.	Pregnenolone sulfate is an endogenous neuroactive steroid with effects on the cognitive functions. It is synthesised in the adrenal glands and the brain by sulfation of pregnenolone, a derivative of cholesterol.	Pre-clinical studies found that treatment with pregnenolone sulfate restores cognitive impairments on gene knockout mice with symptoms of schizophrenia (60, 61). Recent studies found serum levels of pregnenolone sulfate were positively associated with acute ischemic stroke within 24 hours after symptom onset (62) and negatively correlated with menstrually-related migraine (63).

PFOS - Perfluorooctane sulfonate;

*: Indicates a compound that has not been confirmed based on a standard.

Supplementary Figures

Supplementary Figure S1. Mean differences in weight between the high and low BMI genetic risk score groups.

Error bars represent the 95% confidence intervals of mean differences in weight. Sample size ranges from 111 (at age 31 months) to 744 (at age 24 years). Test results are given for a Students (two-sample two-sided) t-test. ***: p-value < 0.001; **: p-value < 0.01; *: p-value < 0.

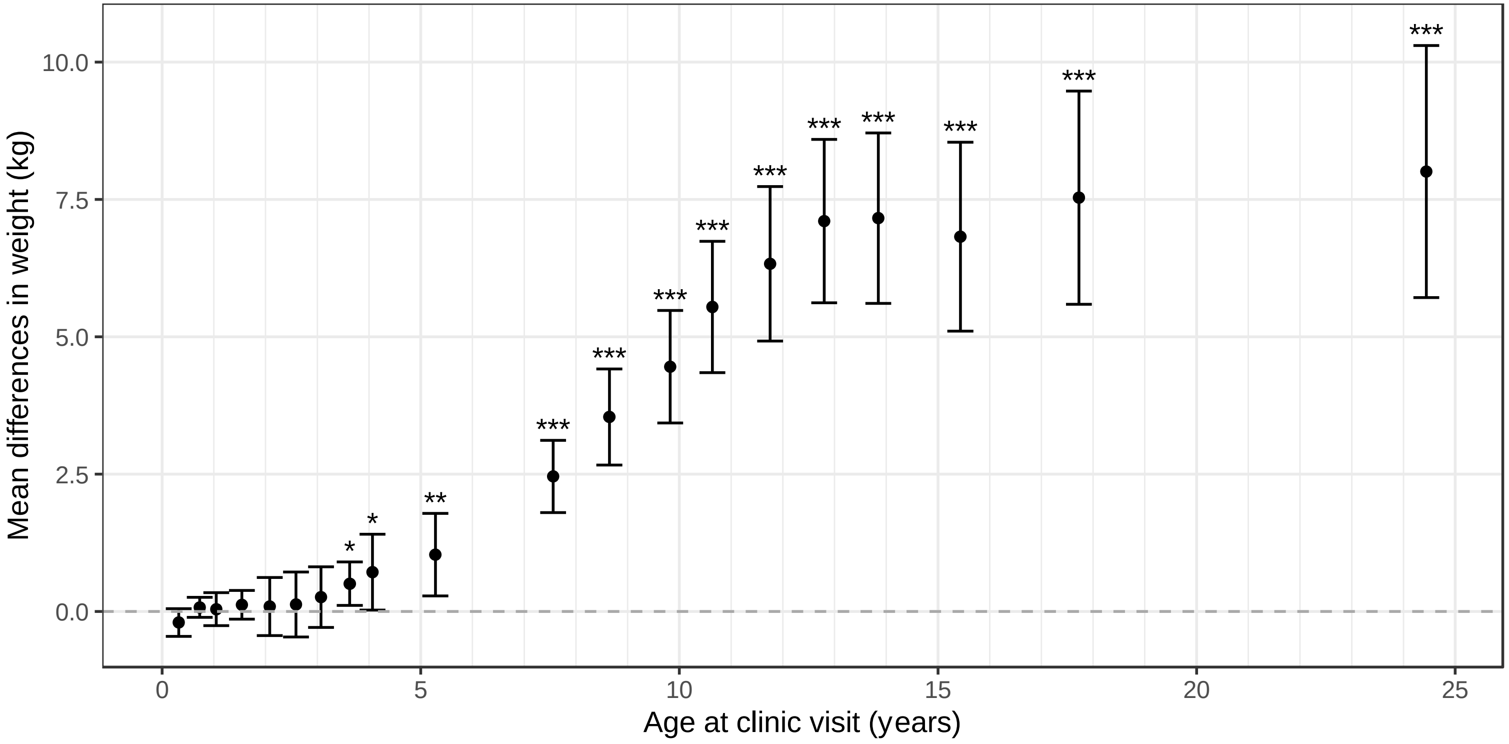

Supplementary Figure S2. Distribution across social class categories by BMI genetic risk score group

BMI = body mass index; GRS = genetic risk score.

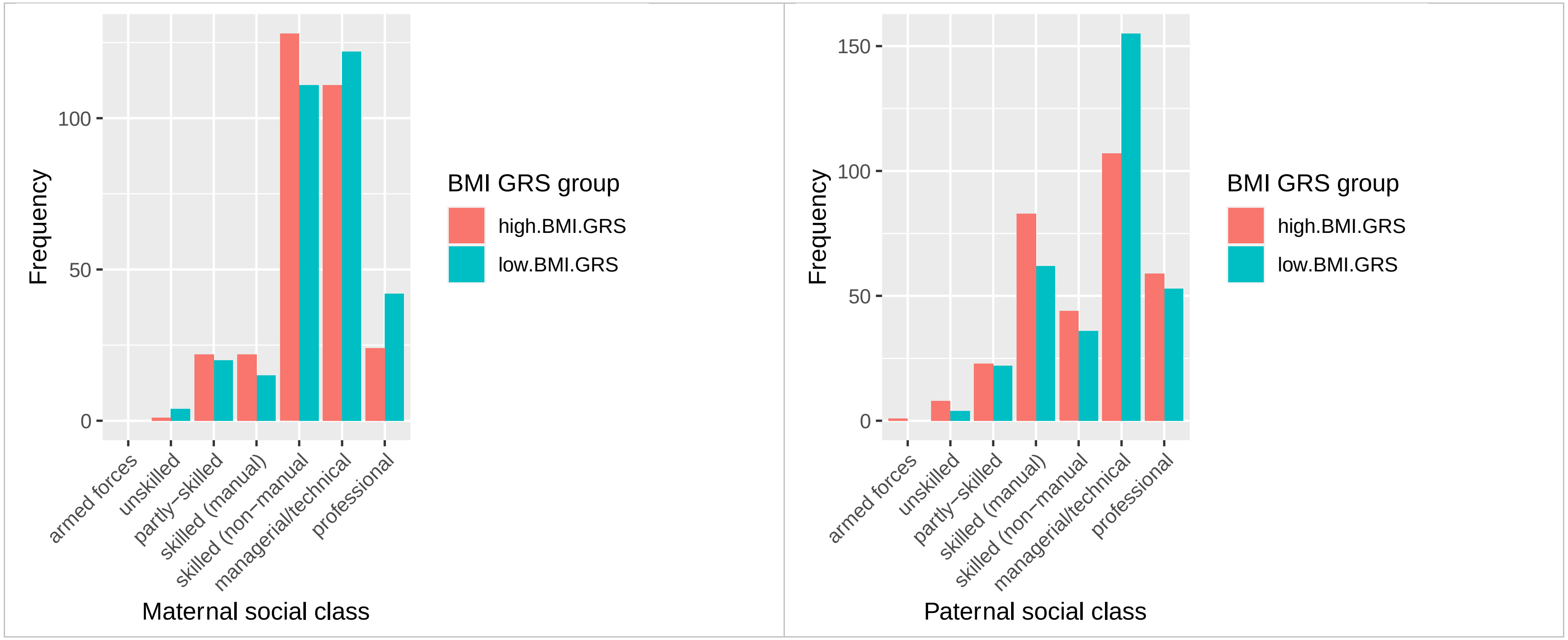

Supplementary Figure S3. Distribution of z-scored levels of BMI genetic risk score group associated metabolites by group.

See separate PDF.

Supplementary Figure S4. Comparison of BMI genetic risk score group effects estimated from Model 1 to BMI effect estimates based on measured BMI

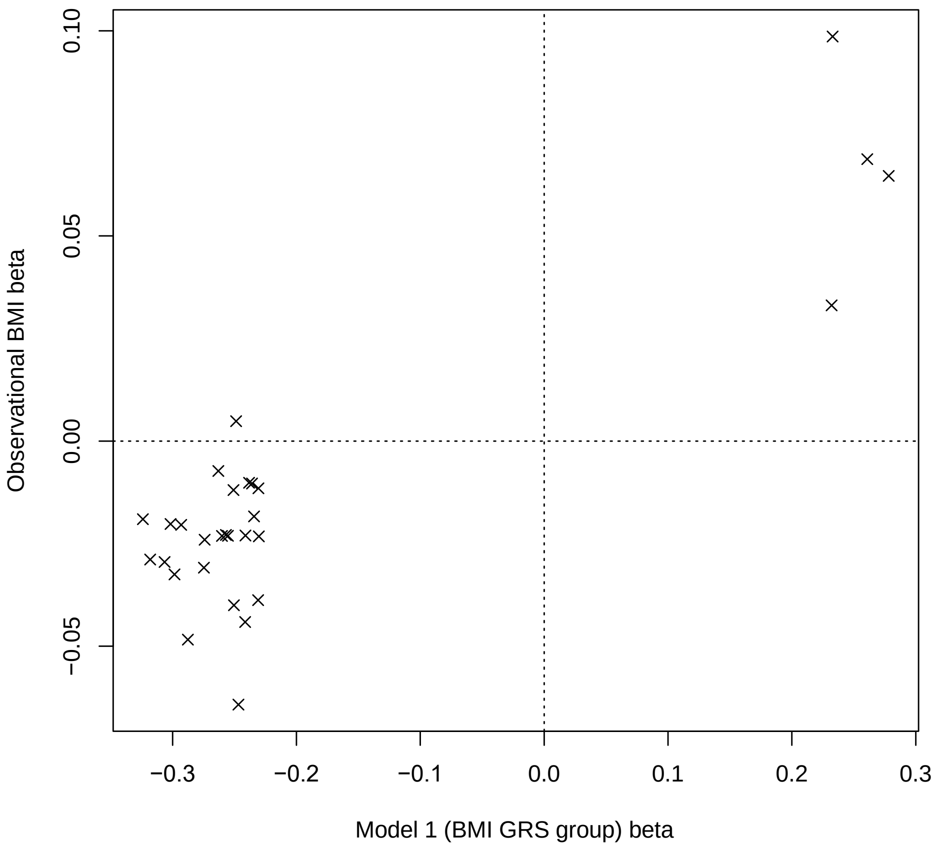

Supplementary Figure S5. Relationship between selected BMI genetic risk score group associated metabolites and measured BMI. Based on measured BMI at the age 24 years clinic. Yellow = low BMI genetic risk score (GRS) group; blue = high BMI GRS group. $\beta_{overall}$ is the measured BMI effect ($CI_{95\%}$ = 95% confidence interval), extracted from multivariate linear model fitted in all individuals [metabolite ~ BMI + BMI.GRS.group + sex + age]. Where an interaction term improved the fit of the model and/or the metabolite level was not associated with measured BMI (p>0.05), the measured BMI effect (adjusted for age and sex) is given for each BMI GRS group separately ($\beta_{BMI\_GRS\_high}$, $\beta_{BMI\_GRS\_low}$). In the plots, solid lines denote the predicted univariate within GRS group relationship between BMI and metabolite with a 95% confidence interval denoted by shading. Metabolites shown in this figure are the eight representative BMI GRS group associated features that are not included in Figure 5 of the main text.

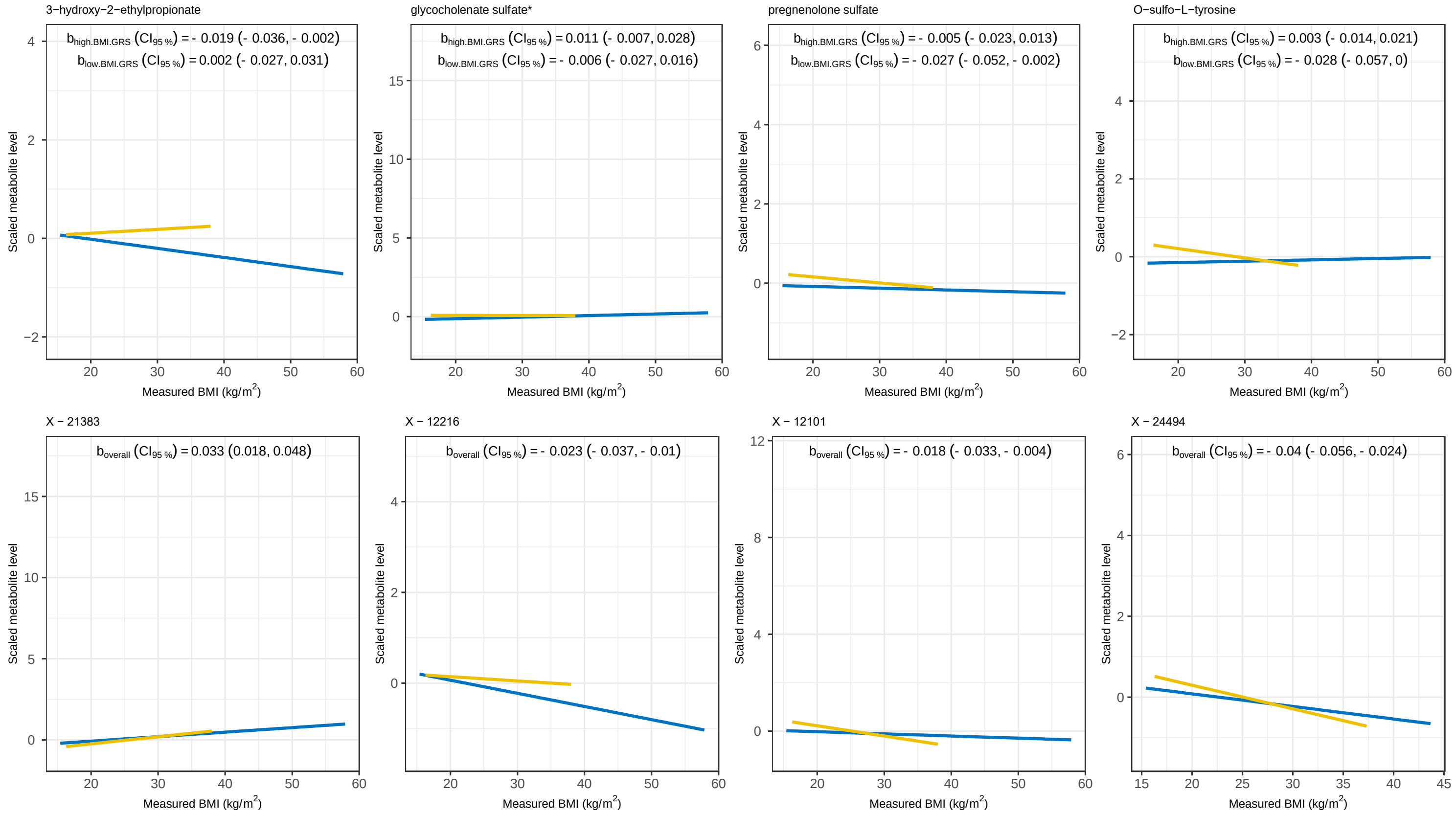

Supplementary References

1. Harris PA, Taylor R, Thielke R, Payne J, Gonzalez N, Conde JG. Research electronic data capture (REDCap)—A metadata-driven methodology and workflow process for providing translational research informatics support. *J Biomed Inform* 2009;42:377–381.
2. Harris PA, Taylor R, Minor BL, *et al.* The REDCap consortium: Building an international community of software platform partners. *J Biomed Inform* 2019;95:103208.
3. ALSPAC. (2020). Explore data and samples. [WWW document]. URL http://www.bristol.ac.uk/alspac/researchers/our-data/
4. ALSPAC Ethics and Law Committee. (2020). Research ethics. [WWW document]. URL http://www.bristol.ac.uk/alspac/researchers/research-ethics/
5. Yengo L, Sidorenko J, Kemper KE, *et al.* Meta-analysis of genome-wide association studies for height and body mass index in approximately 700000 individuals of European ancestry. *Hum Mol Genet* 2018;27:3641–3649.
6. Yang J, Lee SH, Goddard ME, Visscher PM. GCTA: A Tool for Genome-wide Complex Trait Analysis. *Am J Hum Genet* 2011;88:76–82.
7. Purcell S, Neale B, Todd-Brown K, *et al.* PLINK: A Tool Set for Whole-Genome Association and Population-Based Linkage Analyses. *Am J Hum Genet* 2007;81:559–575.
8. Evans AM, DeHaven CD, Barrett T, Mitchell M, Milgram E. Integrated, Nontargeted Ultrahigh Performance Liquid Chromatography/Electrospray Ionization Tandem Mass Spectrometry Platform for the Identification and Relative Quantification of the Small-Molecule Complement of Biological Systems. *Anal Chem* 2009;81:6656–6667.
9. DeHaven CD, Evans AM, Dai H, Lawton KA. Organization of GC/MS and LC/MS metabolomics data into chemical libraries. *J Cheminform* 2010;2:9.
10. Howe LD, Tilling K, Galobardes B, Smith GD, Gunnell D, Lawlor DA. Socioeconomic differences in childhood growth trajectories: At what age do height inequalities emerge? *J Epidemiol Community Health* 2012;66:143–148.
11. Haworth S, Mitchell R, Corbin L, *et al.* Apparent latent structure within the UK Biobank sample has implications for epidemiological analysis. *Nat Commun* 2019;10:333.
12. R Core Team. R: A Language and Environment for Statistical Computing. 2020.
13. Hughes DA, Taylor K, McBride N, *et al.* metaboprep: an R package for preanalysis data description and processing. *Bioinformatics* 2022.
14. Stekhoven DJ, Buhlmann P. MissForest--non-parametric missing value imputation for mixed-type data. *Bioinformatics* 2012;28:112–118.
15. Corbin LJ, Tan VY, Hughes DA, *et al.* Formalising recall by genotype as an efficient approach to detailed phenotyping and causal inference. *Nat Commun* 2018;9:711.
16. Fox J, Weisberg S. *An "R" Companion to Applied Regression*. Third. Thousand Oaks, CA: Sage; 2019.

17. Kang G, Liu W, Cheng C, *et al.* Evaluation of a two-step iterative resampling procedure for internal validation of genome-wide association studies. *J Hum Genet* 2015;60:729.

18. Hughes D. (2020). iPVs. [WWW document]. URL https://github.com/hughesevoanth/iPVs

19. Takei R, Inoue T, Sonoda N, *et al.* Bilirubin reduces visceral obesity and insulin resistance by suppression of inflammatory cytokines. *PLoS One* 2019;14:e0223302.

20. Hinds Jr. TD, Stec DE. Bilirubin Safeguards Cardiorenal and Metabolic Diseases: a Protective Role in Health. *Curr Hypertens Rep* 2019;21:87.

21. Weaver L, Hamoud AR, Stec DE, Hinds Jr. TD. Biliverdin reductase and bilirubin in hepatic disease. *Am J Physiol Gastrointest Liver Physiol* 2018;314:G668–G676.

22. Jenko-Praznikar Z, Petelin A, Jurdana M, Ziberna L. Serum bilirubin levels are lower in overweight asymptomatic middle-aged adults: an early indicator of metabolic syndrome? *Metabolism* 2013;62:976–985.

23. Choi Y, Lee SJ, Spiller W, *et al.* Causal Associations Between Serum Bilirubin Levels and Decreased Stroke Risk. *Arterioscler Thromb Vasc Biol* 2020;40:437–445.

24. Hou L, Li H, Si S, *et al.* Exploring the causal pathway from bilirubin to CVD and diabetes in the UK biobank cohort study: Observational findings and Mendelian randomization studies. *Atherosclerosis* 2021;320:112–121.

25. Chen G, Adeyemo A, Zhou J, *et al.* A UGT1A1 variant is associated with serum total bilirubin levels, which are causal for hypertension in African-ancestry individuals. *npj Genomic Med* 2021;6:44.

26. Stender S, Frikke-Schmidt R, Nordestgaard BG, Tybjærg-Hansen A. Extreme Bilirubin Levels as a Causal Risk Factor for Symptomatic Gallstone Disease. *JAMA Intern Med* 2013;173:1222–1228.

27. Seyed Khoei N, Jenab M, Murphy N, *et al.* Circulating bilirubin levels and risk of colorectal cancer: serological and Mendelian randomization analyses. *BMC Med* 2020;18:229.

28. Abbasi A, Deetman PE, Corpeleijn E, *et al.* Bilirubin as a potential causal factor in type 2 diabetes risk: A mendelian randomization study. *Diabetes* 2015;64:1459–1469.

29. Slotte PJ. Biological functions of sphingomyelins. *Prog Lipid Res* 2013;52:424–437.

30. Slotte PJ. Molecular properties of various structurally defined sphingomyelins – Correlation of structure with function. *Prog Lipid Res* 2013;52:206–219.

31. Jiang X, Paultre F, Pearson TA, *et al.* Plasma Sphingomyelin Level as a Risk Factor for Coronary Artery Disease. *Arterioscler Thromb Vasc Biol* 2000;20:2614–2618.

32. Schlitt A, Blankenberg S, Yan D, *et al.* Further evaluation of plasma sphingomyelin levels as a risk factor for coronary artery disease. *Nutr Metab (Lond)* 2006;3:5.

33. Lees HJ, Swann JR, Wilson ID, Nicholson JK, Holmes E. Hippurate: the natural history of a mammalian-microbial cometabolite. *J Proteome Res* 2013;12:1527–1546.

34. Pallister T, Jackson MA, Martin TC, *et al.* Untangling the relationship between diet and visceral fat mass through blood metabolomics and gut microbiome profiling. *Int J*

Obes 2017;41:1106–1113.

35. Pallister T, Jackson MA, Martin TC, *et al.* Hippurate as a metabolomic marker of gut microbiome diversity: Modulation by diet and relationship to metabolic syndrome. *Sci Rep* 2017;7:13670.

36. otto L, Budde K, Kastenmüller G, *et al.* Associations between adipose tissue volume and small molecules in plasma and urine among asymptomatic subjects from the general population. 2020.

37. Buck RC, Franklin J, Berger U, *et al.* Perfluoroalkyl and polyfluoroalkyl substances in the environment: Terminology, classification, and origins. *Integr Environ Assess Manag* 2011;7:513–541.

38. Environment Agency. *Perfluorooctane sulfonate (PFOS) and related substances: sources, pathways and environmental data*. Bristol; 2019.

39. Domingo JL, Nadal M. Human exposure to per- and polyfluoroalkyl substances (PFAS) through drinking water: A review of the recent scientific literature. *Environ Res* 2019;177:108648.

40. Tittlemier SA, Pepper K, Seymour C, *et al.* Dietary Exposure of Canadians to Perfluorinated Carboxylates and Perfluorooctane Sulfonate via Consumption of Meat, Fish, Fast Foods, and Food Items Prepared in Their Packaging. *J Agric Food Chem* 2007;55:3203–3210.

41. Ericson I, Martí-Cid R, Nadal M, Van Bavel B, Lindström G, Domingo JL. Human Exposure to Perfluorinated Chemicals through the Diet: Intake of Perfluorinated Compounds in Foods from the Catalan (Spain) Market. *J Agric Food Chem* 2008;56:1787–1794.

42. Noorlander CW, van Leeuwen SPJ, te Biesebeek JD, Mengelers MJB, Zeilmaker MJ. Levels of Perfluorinated Compounds in Food and Dietary Intake of PFOS and PFOA in The Netherlands. *J Agric Food Chem* 2011;59:7496–7505.

43. Kedikoglou K, Costopoulou D, Vassiliadou I, Leondiadis L. Preliminary assessment of general population exposure to perfluoroalkyl substances through diet in Greece. *Environ Res* 2019;177:108617.

44. Jian J-M, Chen D, Han F-J, *et al.* A short review on human exposure to and tissue distribution of per- and polyfluoroalkyl substances (PFASs). *Sci Total Environ* 2018;636:1058–1069.

45. Pérez F, Nadal M, Navarro-Ortega A, *et al.* Accumulation of perfluoroalkyl substances in human tissues. *Environ Int* 2013;59:354–362.

46. Zeng Z, Song B, Xiao R, *et al.* Assessing the human health risks of perfluorooctane sulfonate by in vivo and in vitro studies. *Environ Int* 2019;126:598–610.

47. Harada K, Saito N, Inoue K, *et al.* The influence of time, sex and geographic factors on levels of perfluorooctane sulfonate and perfluorooctanoate in human serum over the last 25 years. *J Occup Health* 2004;46:141–147.

48. Perogamvros I, Ray DW, Trainer PJ. Regulation of cortisol bioavailability—effects on hormone measurement and action. *Nat Rev Endocrinol* 2012;8:717–727.

49. Björntorp P, Rosmond R. Obesity and cortisol. *Nutrition* 2000;16:924–936.

50. Abraham SB, Rubino D, Sinaii N, Ramsey S, Nieman LK. Cortisol, obesity, and the metabolic syndrome: A cross-sectional study of obese subjects and review of the literature. *Obesity* 2013;21:E105–E117.

51. Sekula P, Goek O-N, Quaye L, *et al.* A Metabolome-Wide Association Study of Kidney Function and Disease in the General Population. *J Am Soc Nephrol* 2016;27:1175.

52. Velenosi TJ, Thomson BKA, Tonial NC, *et al.* Untargeted metabolomics reveals N, N, N-trimethyl-L-alanyl-L-proline betaine (TMAP) as a novel biomarker of kidney function. *Sci Rep* 2019;9:6831.

53. Lustgarten MS, Price LL, Chale A, Phillips EM, Fielding RA. Branched Chain Amino Acids Are Associated With Muscle Mass in Functionally Limited Older Adults. *Journals Gerontol Ser A* 2013;69:717–724.

54. Alonso A, Yu B, Qureshi WT, *et al.* Metabolomics and Incidence of Atrial Fibrillation in African Americans: The Atherosclerosis Risk in Communities (ARIC) Study. *PLoS One* 2015;10:e0142610.

55. Alonso A, Yu B, Sun Y V, *et al.* Serum Metabolomics and Incidence of Atrial Fibrillation (from the Atherosclerosis Risk in Communities Study). *Am J Cardiol* 2019;123:1955–1961.

56. Huang J, Weinstein SJ, Kitahara CM, Karoly ED, Sampson JN, Albanes D. A prospective study of serum metabolites and glioma risk. *Oncotarget* 2017;8:70366–70377.

57. Das SK, Ainsworth HC, Dimitrov L, *et al.* Metabolomic architecture of obesity implicates metabolonic lactone sulfate in cardiometabolic disease. *Mol Metab* 2021;54:101342.

58. Lind L. The metabolomic profile of carotid artery intima-media thickness and echogenicity. *Atherosclerosis* 2021;335:142–147.

59. Wang Z, Xu X, He B, *et al.* The impact of chronic environmental metal and benzene exposure on human urinary metabolome among Chinese children and the elderly population. *Ecotoxicol Environ Saf* 2019;169:232–239.

60. Wong P, Sze Y, Chang CCR, Lee J, Zhang X. Pregnenolone sulfate normalizes schizophrenia-like behaviors in dopamine transporter knockout mice through the AKT/GSK3β pathway. *Transl Psychiatry* 2015;5:e528–e528.

61. Rajagopal L, Soni D, Meltzer HY. Neurosteroid pregnenolone sulfate, alone, and as augmentation of lurasidone or tandospirone, rescues phencyclidine-induced deficits in cognitive function and social interaction. *Behav Brain Res* 2018;350:31–43.

62. Tiedt S, Brandmaier S, Kollmeier H, *et al.* Circulating Metabolites Differentiate Acute Ischemic Stroke from Stroke Mimics. *Ann Neurol* 2020;88:736–746.

63. Rustichelli C, Bellei E, Bergamini S, *et al.* Comparison of pregnenolone sulfate, pregnanolone and estradiol levels between patients with menstrually-related migraine and controls: an exploratory study. *J Headache Pain* 2021;22:13.
